# Supplementary material for: Targeted degradation of zDHHC-PATs decreases substrate S-palmitoylation
Source: PLoS One. 2024 Mar 21;19(3):e0299665. doi: 10.1371/journal.pone.0299665 (PMC10956751; doi:10.1371/journal.pone.0299665)

Figure 1: LiCOR Odyssey FC using chemiluminescence

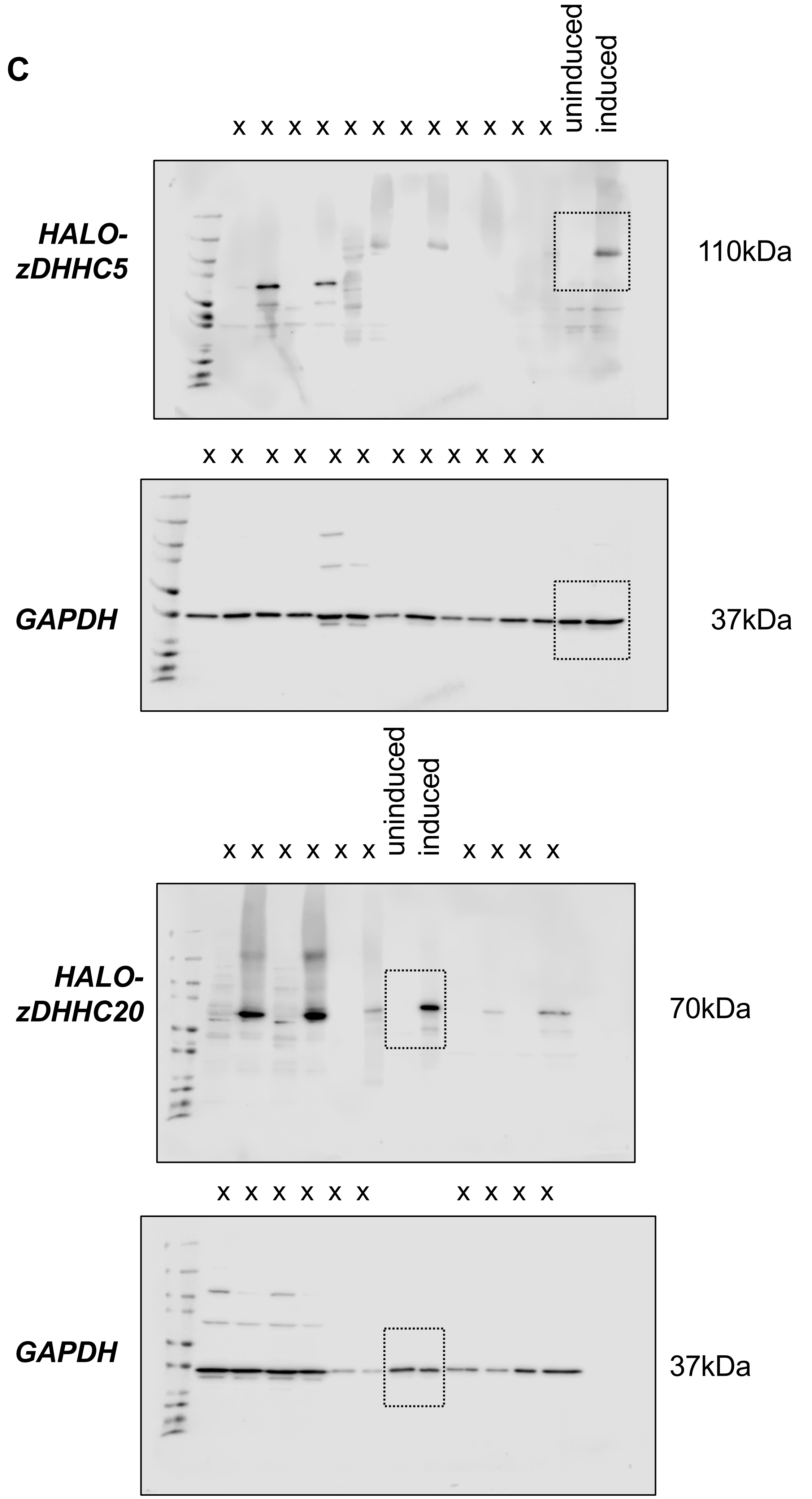

Figure 2: LiCOR Odyssey FC using chemiluminescence

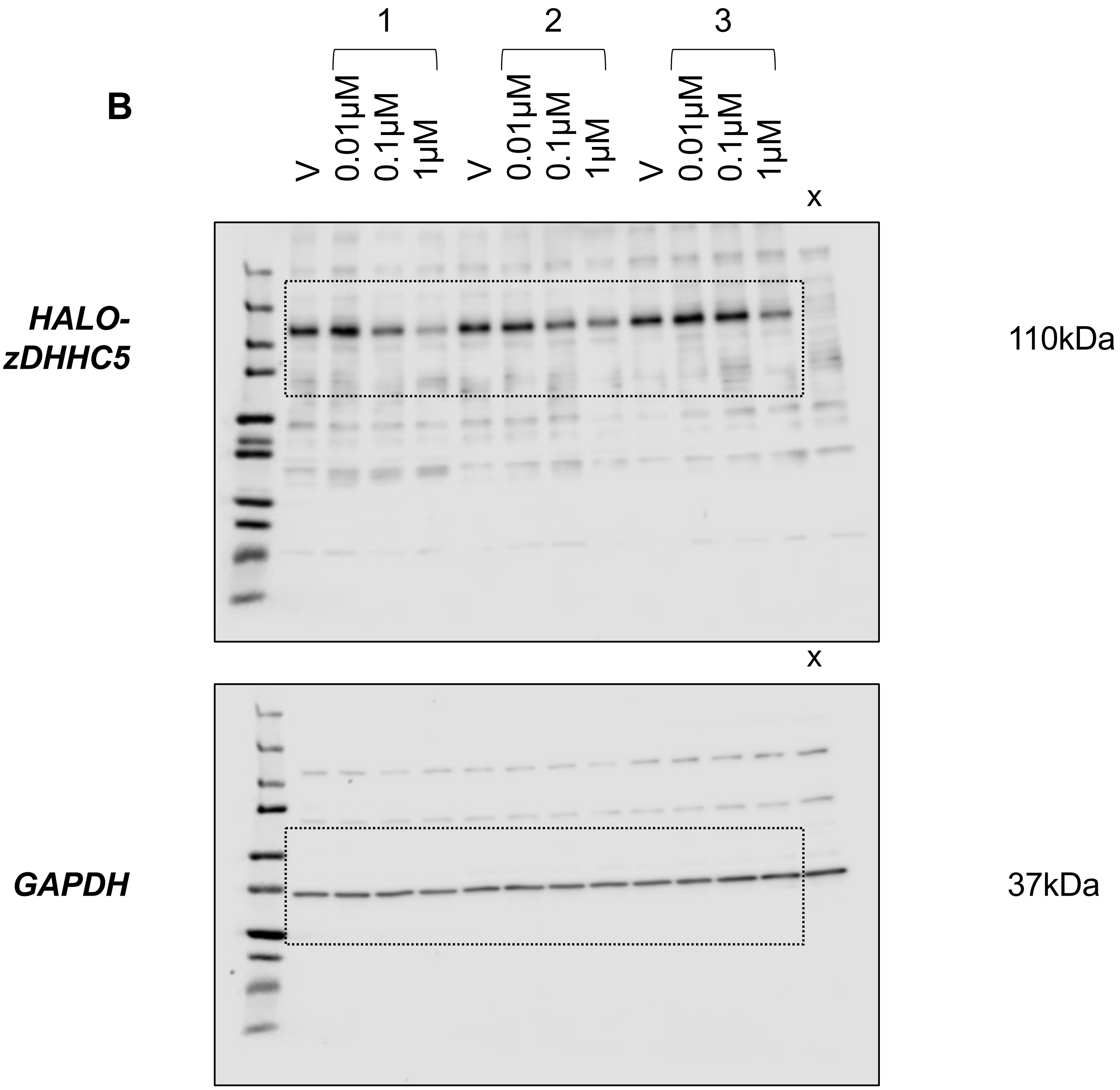

Figure 2: LiCOR Odyssey FC using chemiluminescence

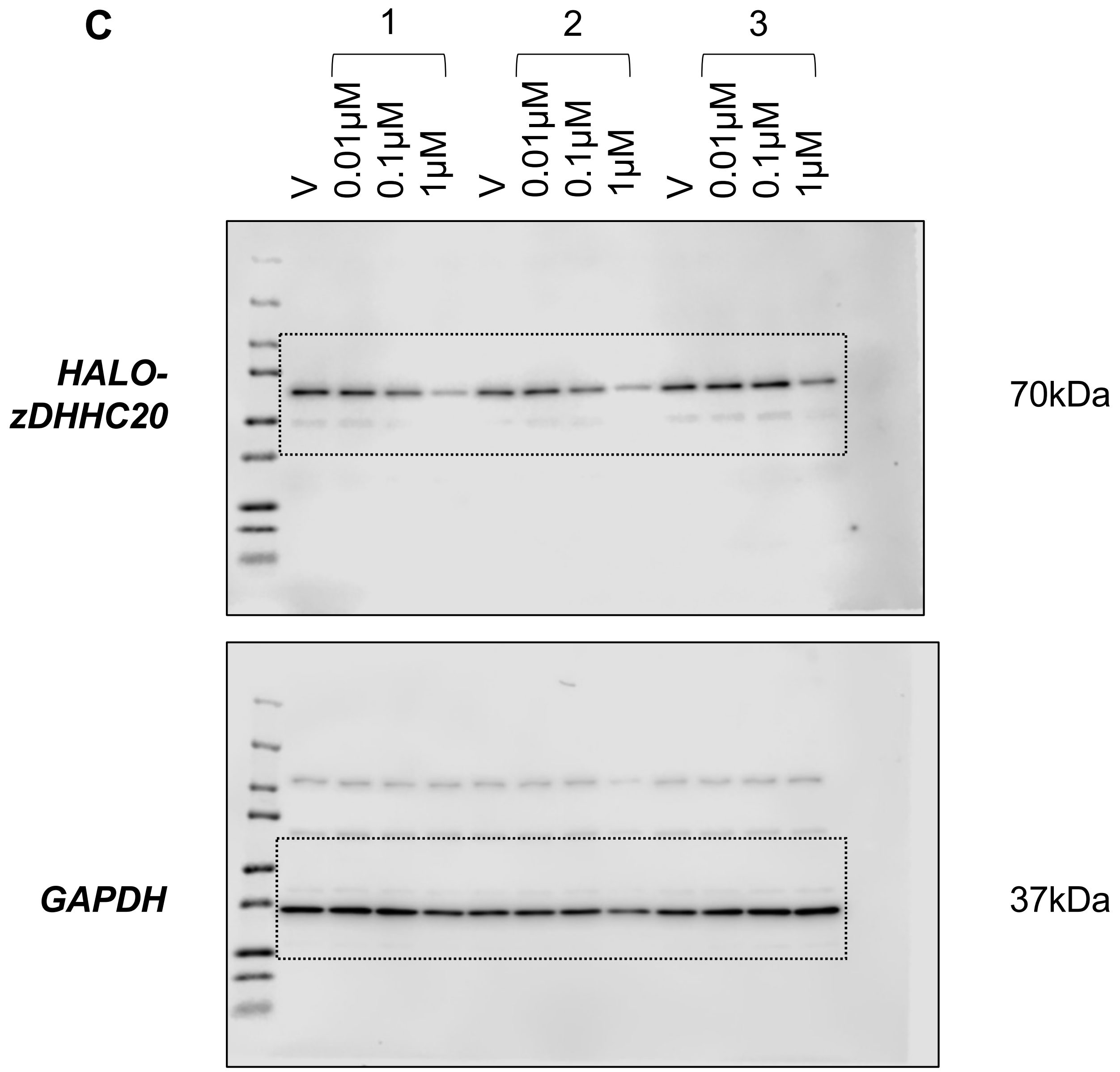

Figure 2: LiCOR Odyssey FC using chemiluminescence

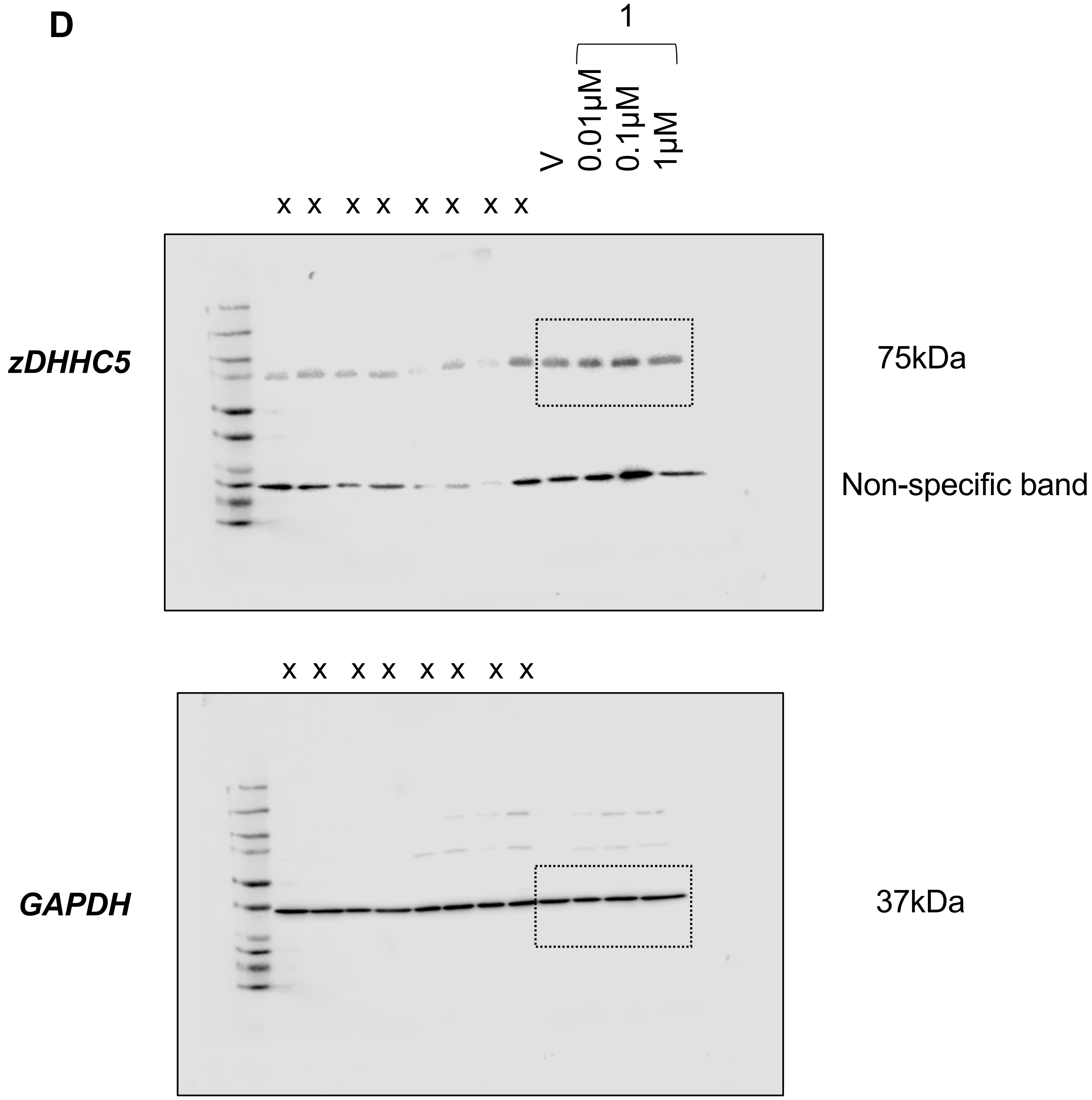

Figure 3: LiCOR Odyssey FC using chemiluminescence

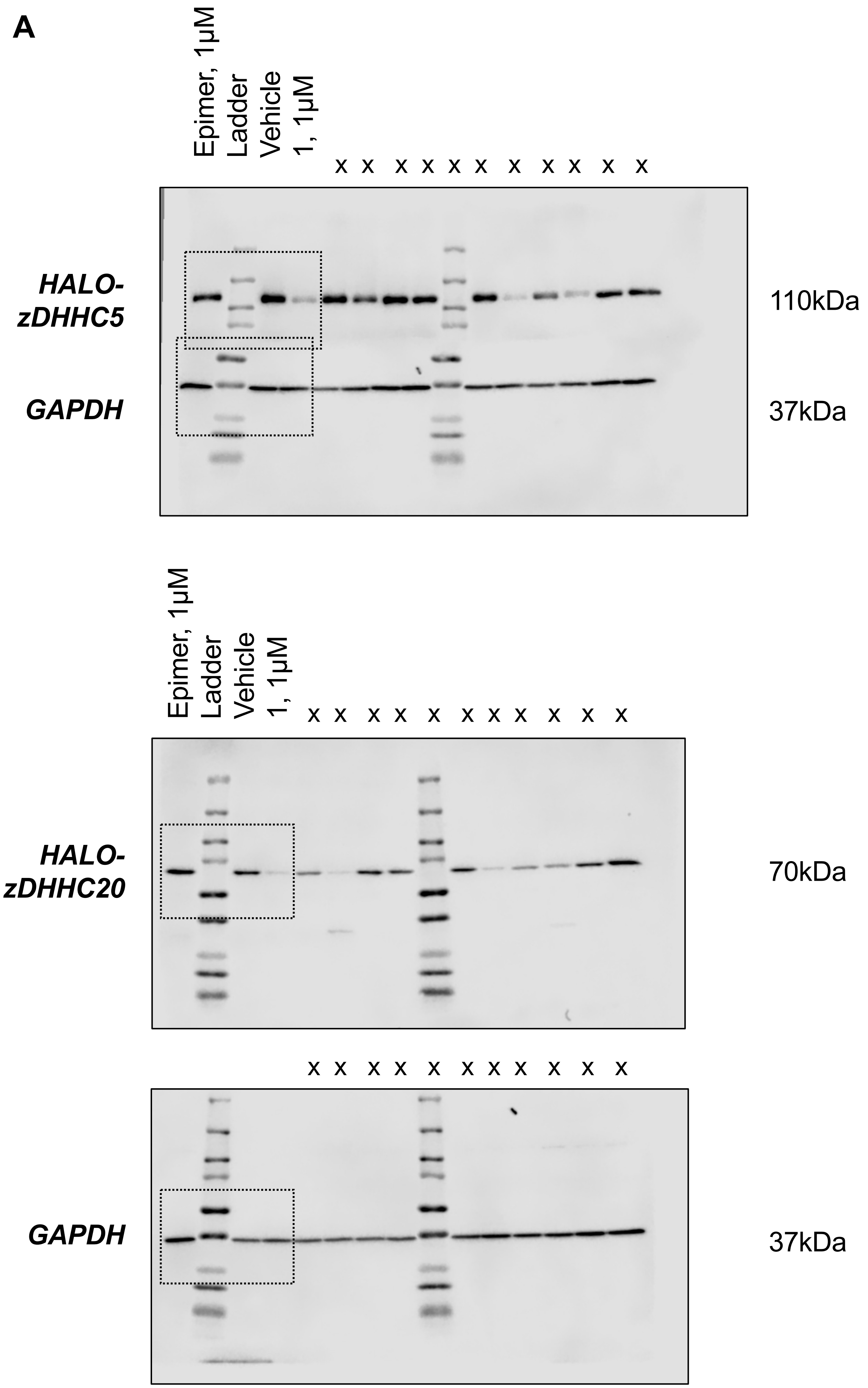

Figure 3: LiCOR Odyssey FC using chemiluminescence

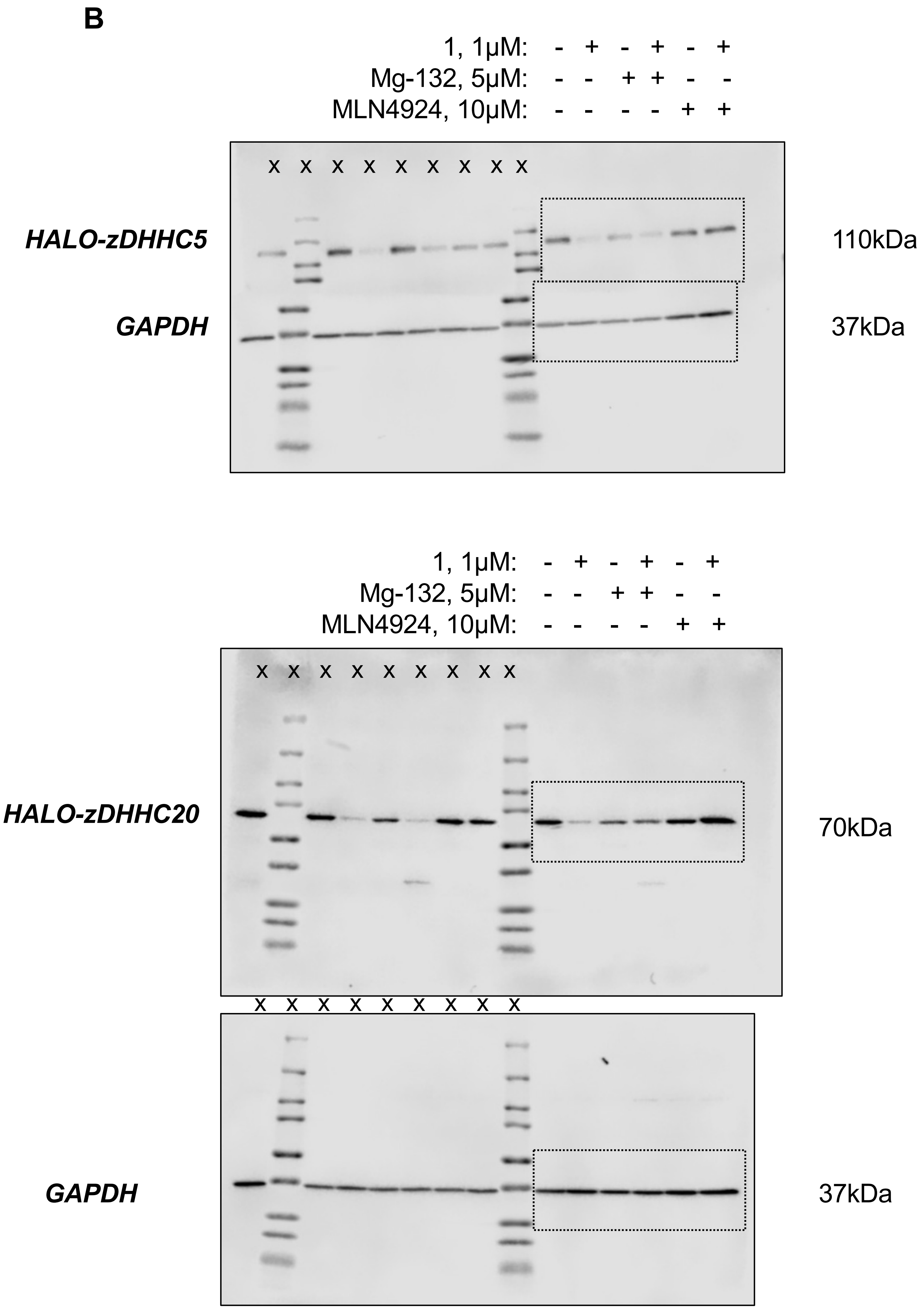

Figure 3: LiCOR Odyssey FC using chemiluminescence

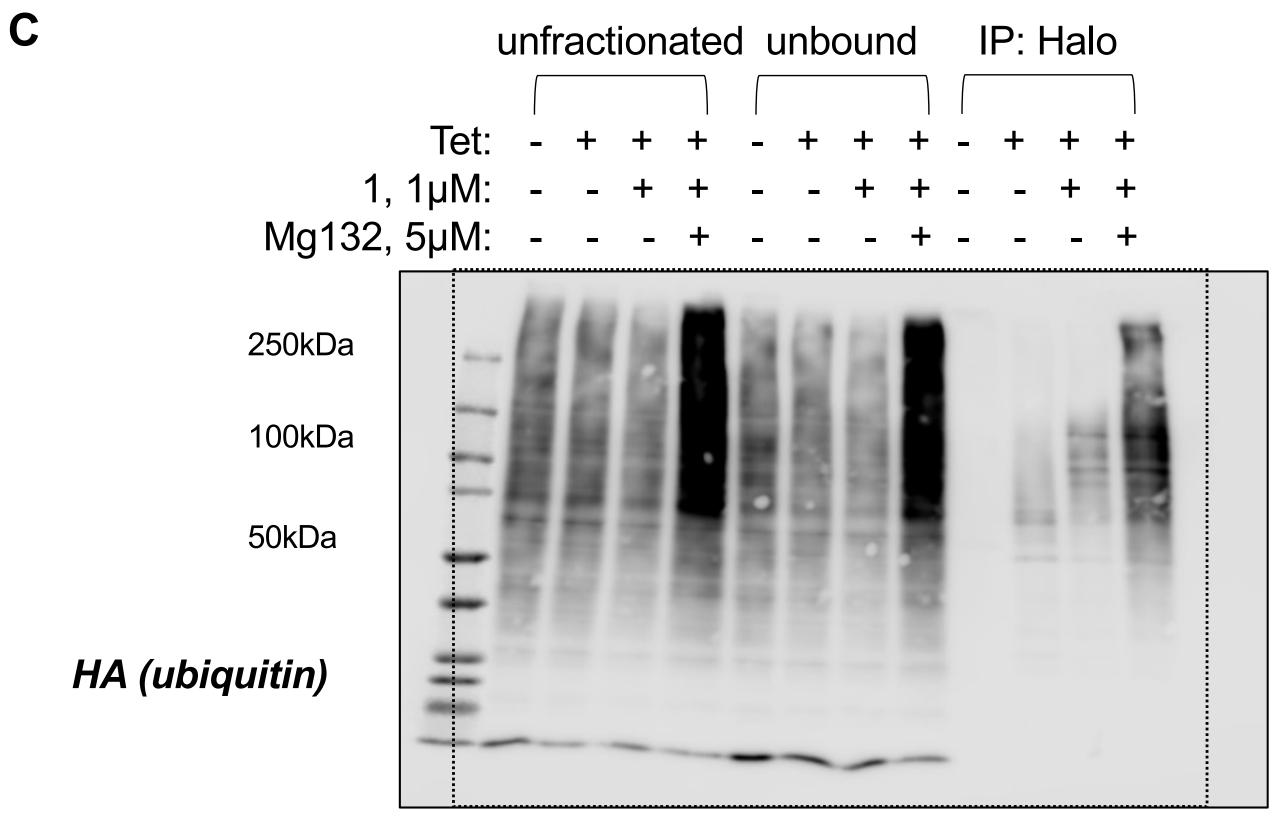

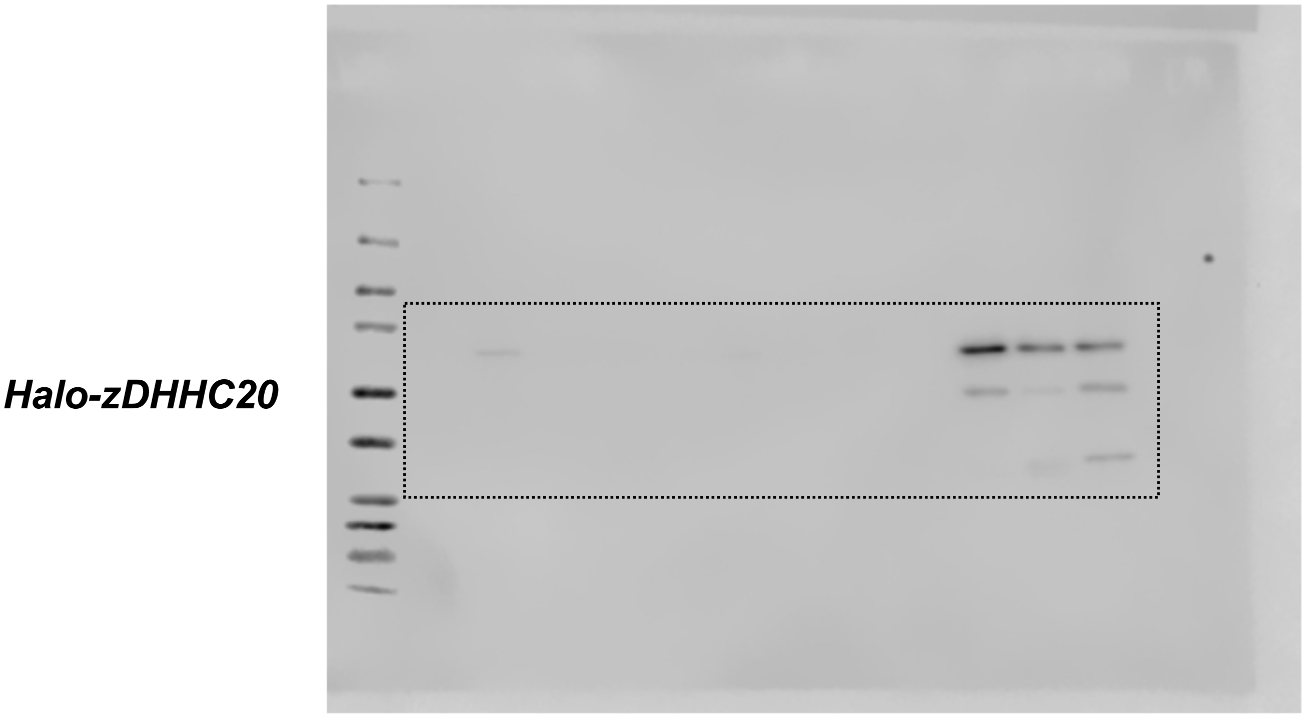

Figure 4: LiCOR Odyssey FC using chemiluminescence

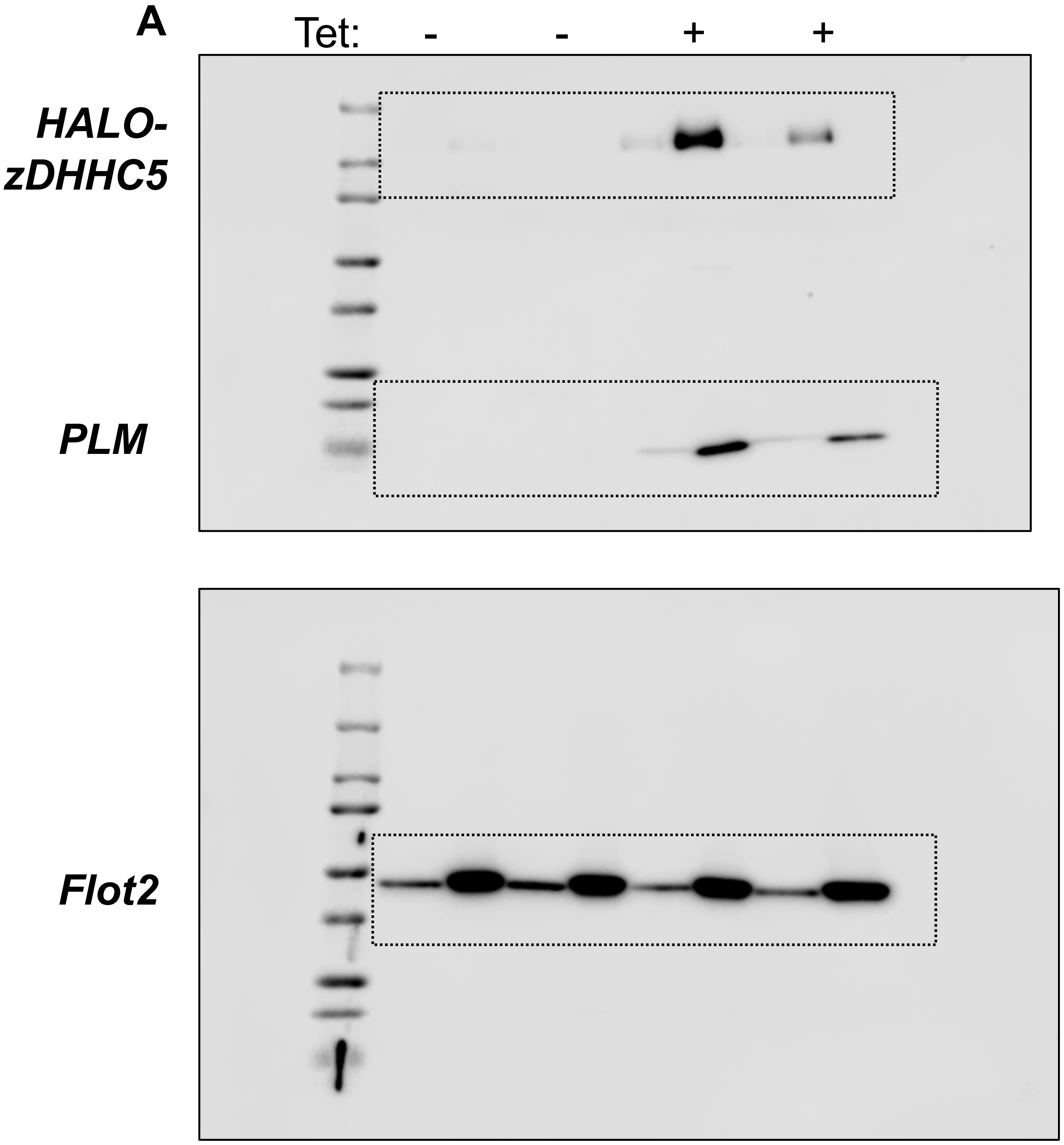

Figure 4: LiCOR Odyssey FC using chemiluminescence

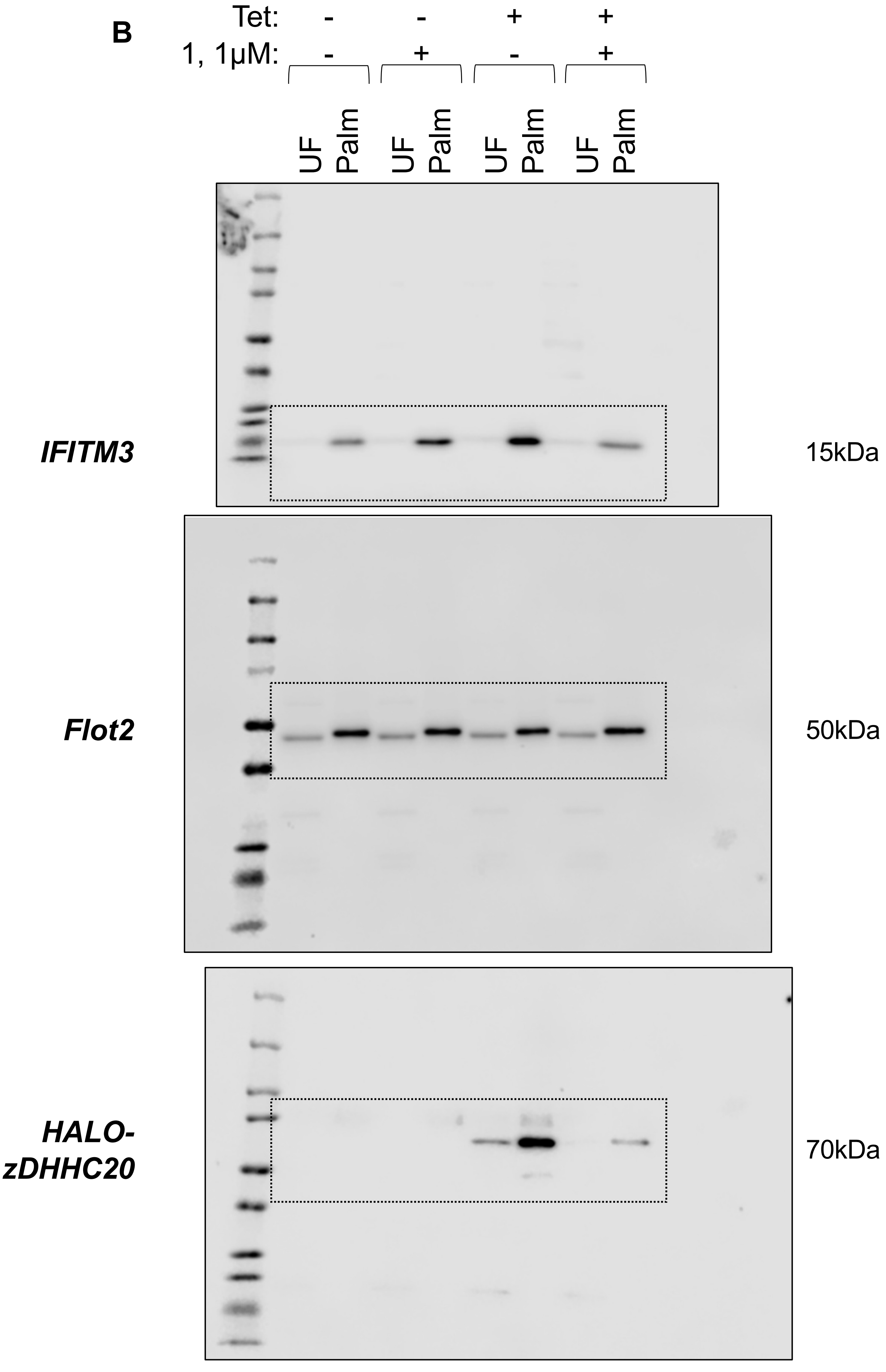

Figure 4: LiCOR Odyssey FC using chemiluminescence

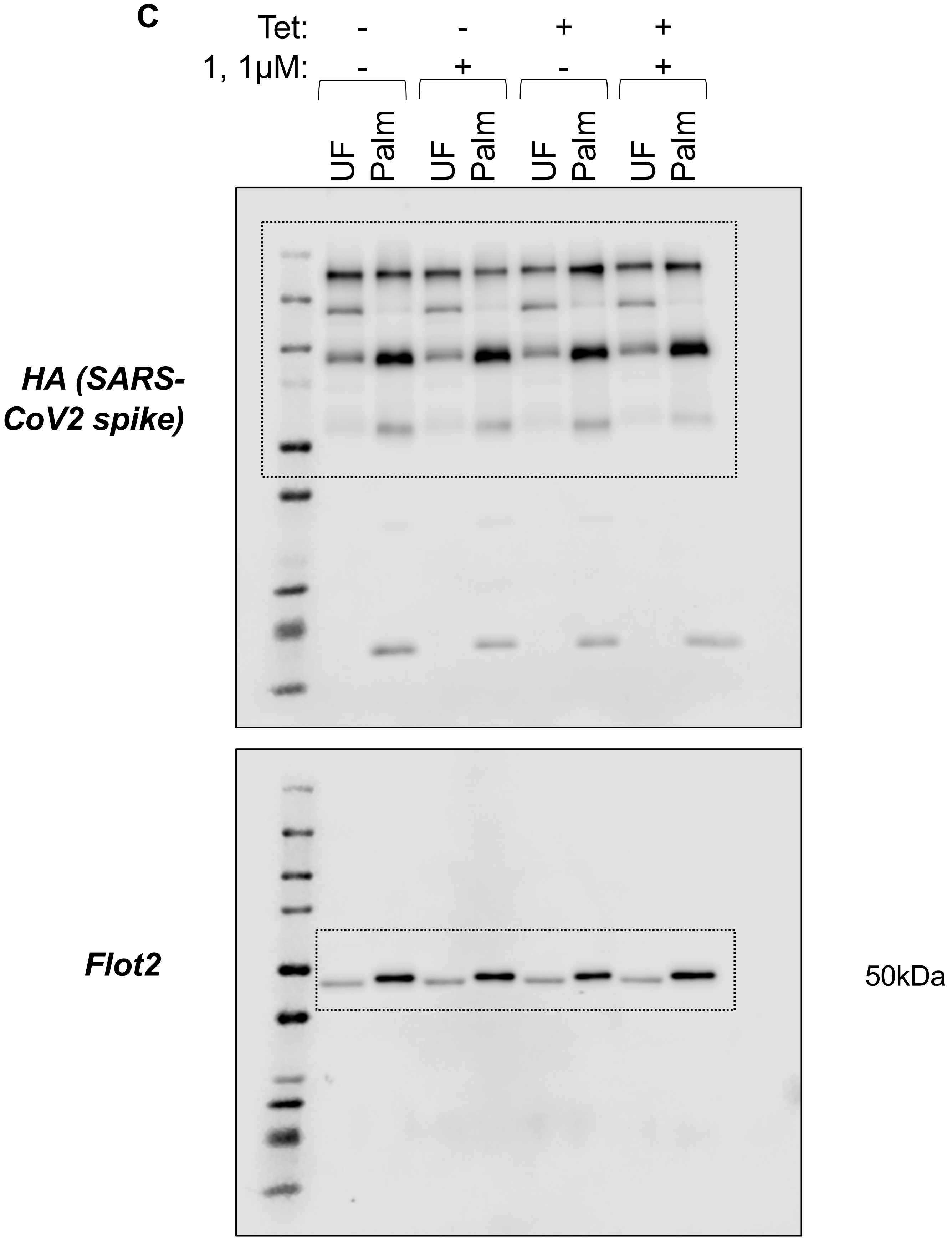

Figure 4: LiCOR Odyssey FC using chemiluminescence

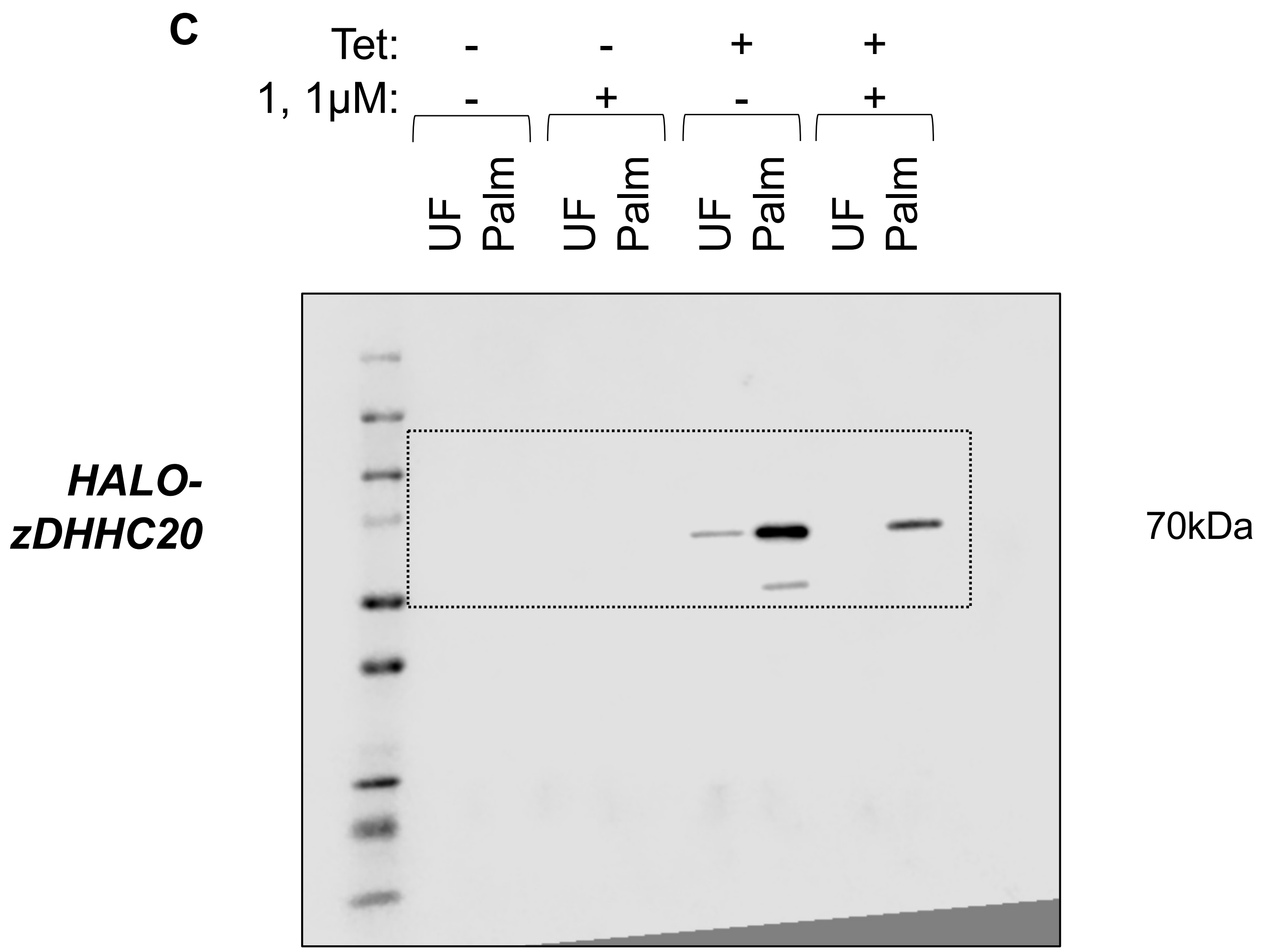

Figure 5: LiCOR Odyssey FC using chemiluminescence

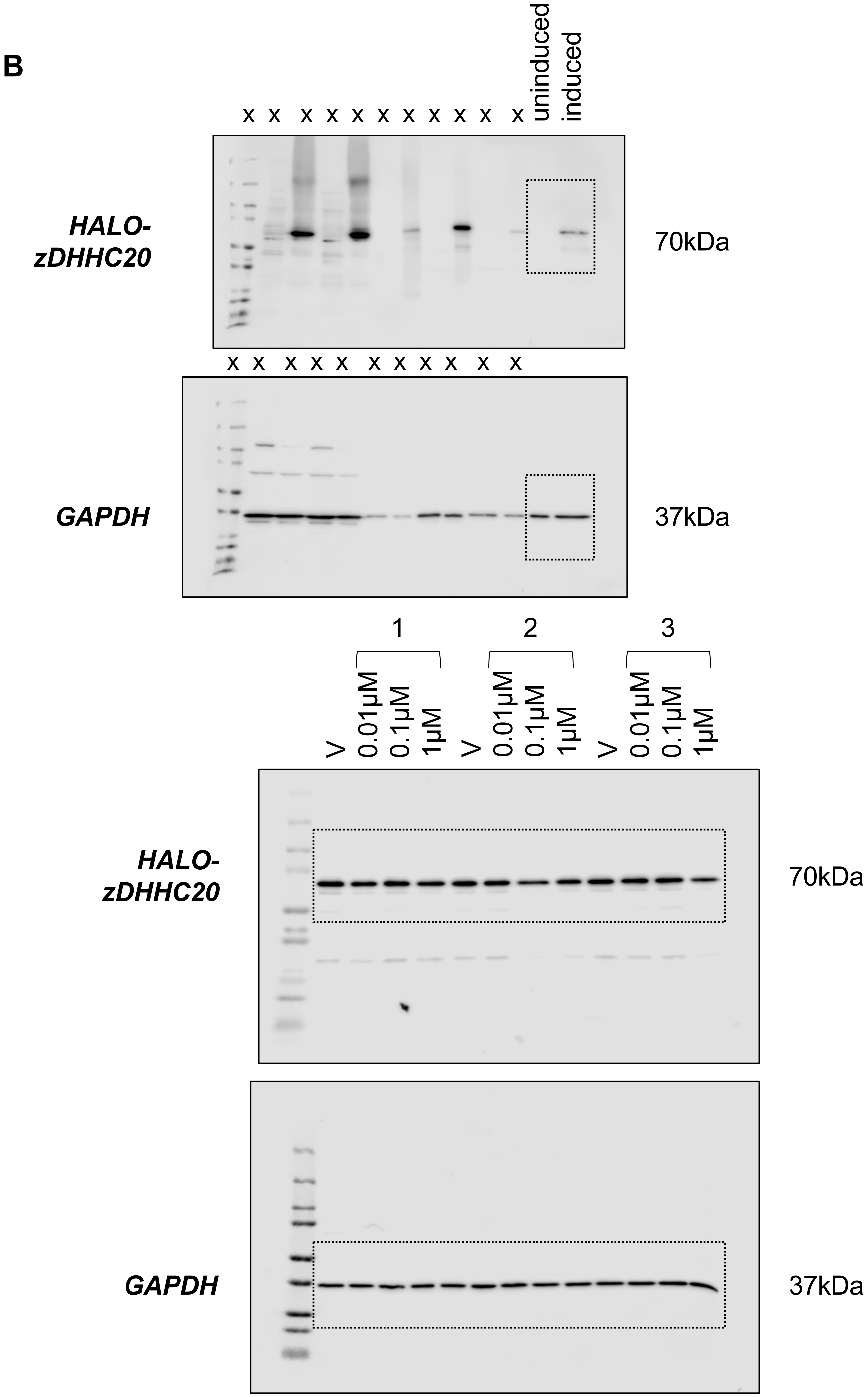

Figure 5: LiCOR Odyssey FC using chemiluminescence

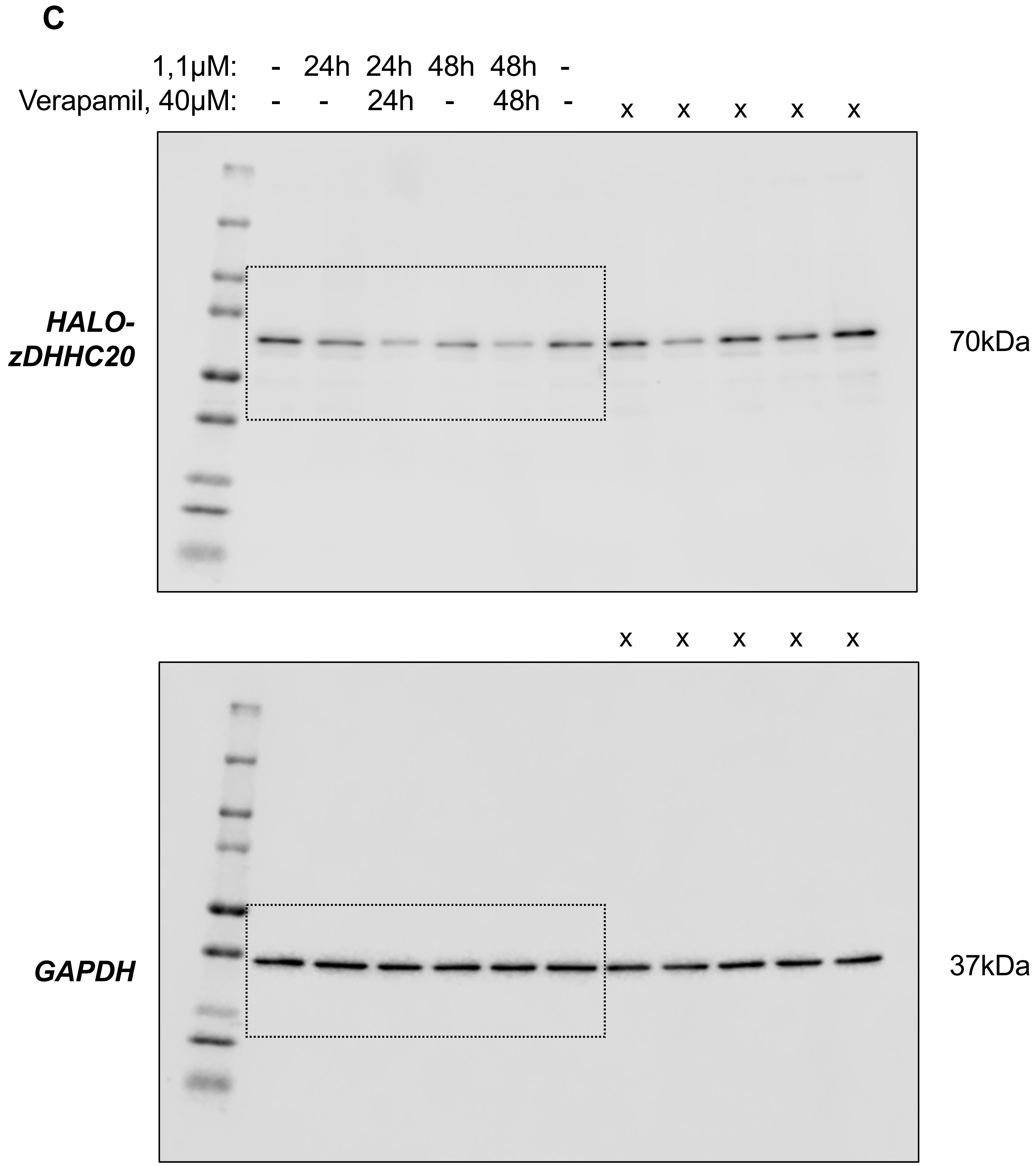

Figure 5: LiCOR Odyssey FC using chemiluminescence

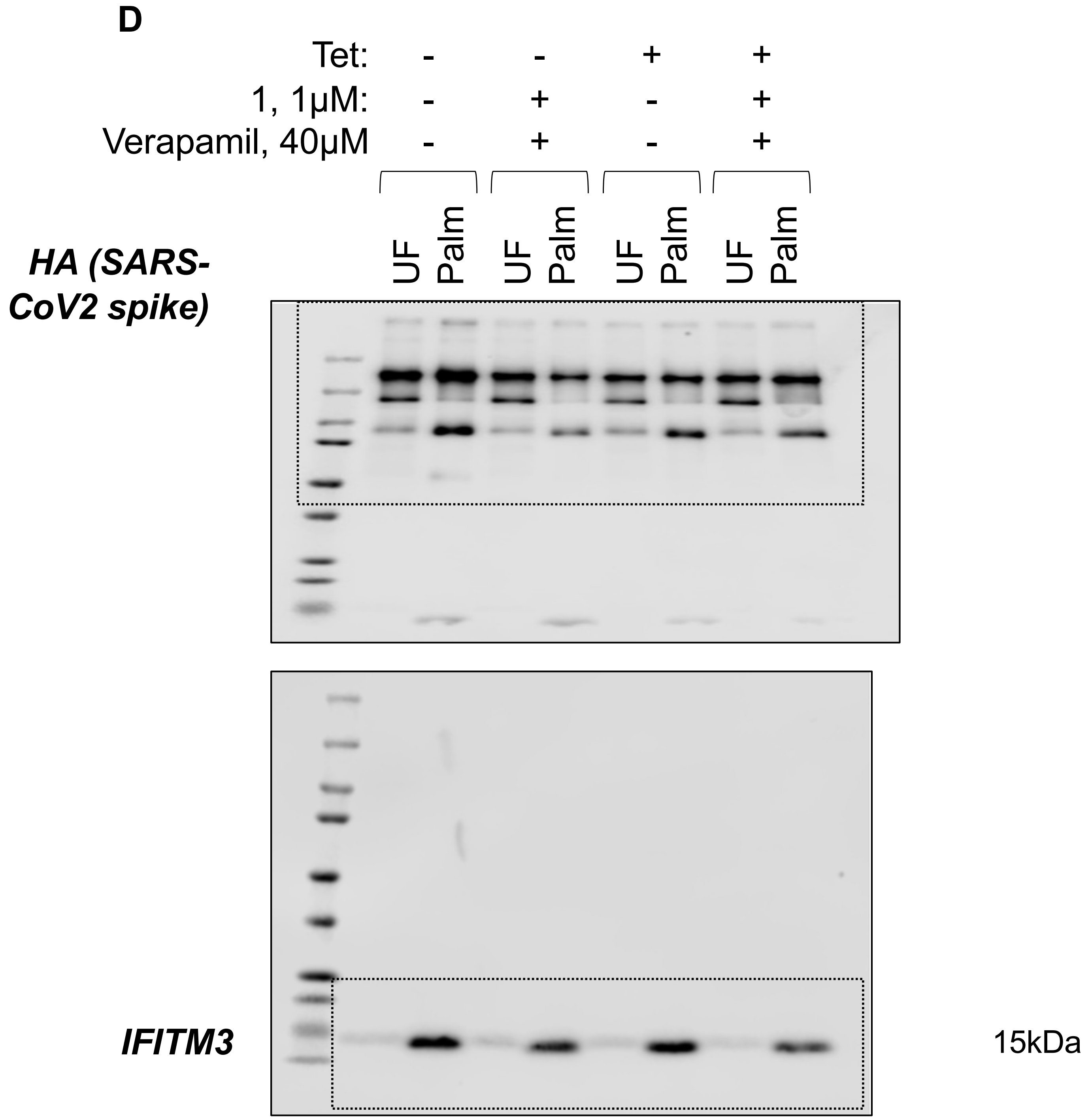

Figure 5: LiCOR Odyssey FC using chemiluminescence

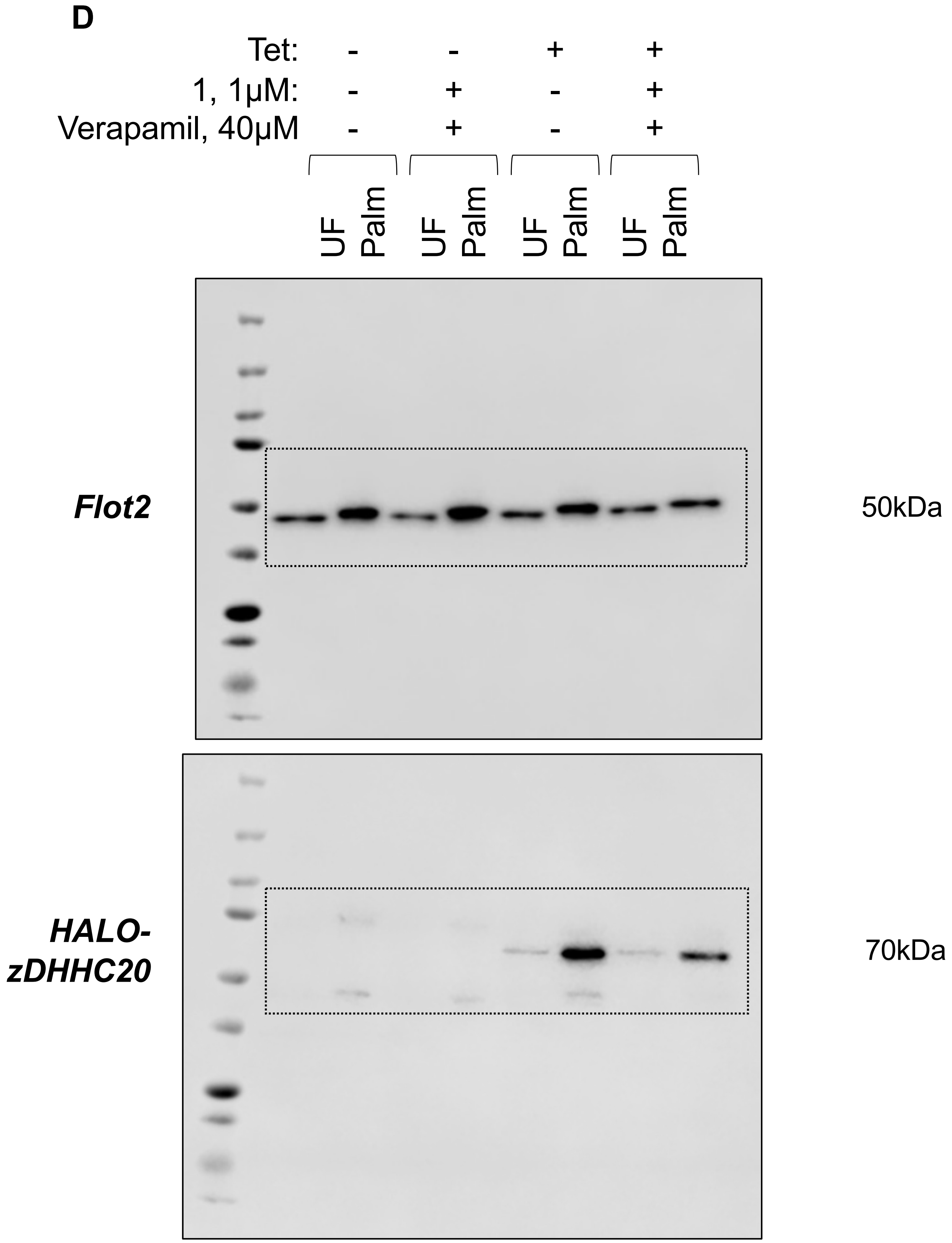

Figure 6: LiCOR Odyssey FC using chemiluminescence

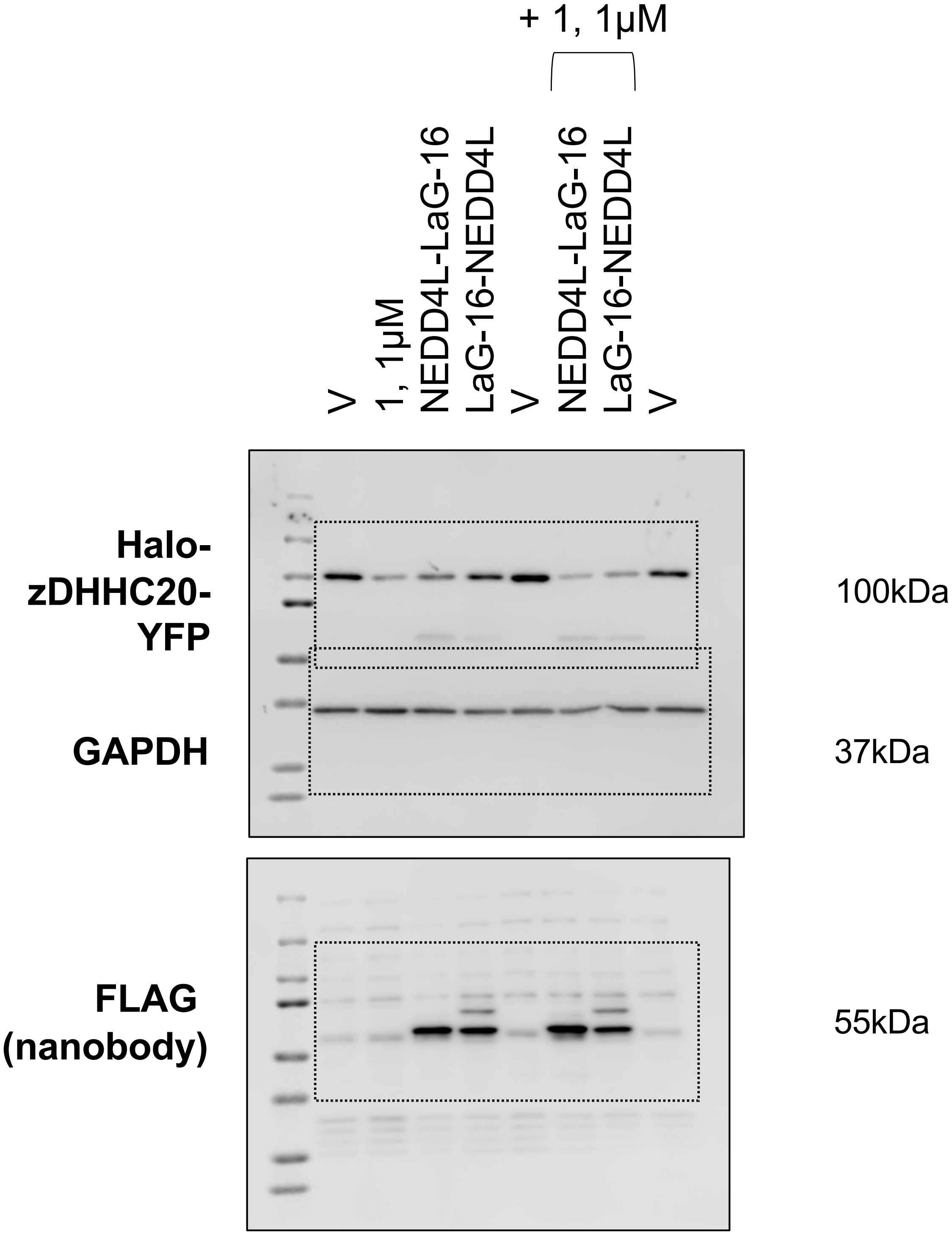

Supplement: S1 Raw images — (PDF) [file pone.0299665.s001.pdf]
