# Supplementary material for: Targeted degradation of zDHHC-PATs decreases substrate S-palmitoylation
Source: PLoS One. 2024 Mar 21;19(3):e0299665. doi: 10.1371/journal.pone.0299665 (PMC10956751; doi:10.1371/journal.pone.0299665)
Supplement: S1 File — Synthesis and evaluation of Halo-PROTACs. (DOCX) [file pone.0299665.s002.docx]

General chemistry experimental details: All reagents were purchased from commercial suppliers and used without further purification unless otherwise stated. Reactions requiring air-sensitive reagents and dry solvents were performed in glassware that had been dried in an oven at 150 °C or flame-dried in vacuo prior to use. These reactions were carried out under argon atmosphere with the exclusion of air. Reactions were monitored by thin-layer chromatography (TLC) on Merck silica gel 60 covered aluminium sheets. TLC plates were visualised under UV-light, and where required with an acidic ethanolic anisaldehyde solution or a KMnO_4_ solution. NMR spectra were recorded on a Bruker DPX-400 spectrometer (^1^H NMR at 400 MHz, ^13^C NMR at 101 MHz and ^19^F NMR at 377 MHz) or a Bruker DPX-500 spectrometer (^1^H NMR at 500 MHz and ^13^C NMR at 126 MHz). Chemical shifts are reported in ppm. ^1^H NMR spectra were recorded with chloroform-d, methanol-d_4_ or DMSO-d_6_ as the solvent using residual CHCl_3_ (δ = 7.26), or CHD_2_OD (δ = 3.31) or (CHD­_2_)SOCD_3_ (δ = 2.50) as internal standard, and for ^13^C NMR spectra the chemical shifts are reported relative to the central resonance of CDCl_3_ (δ = 77.16), CD_3_OD (δ = 49.00) or (CD­_3_)_2_SO (δ = 39.52). Signals in the obtained spectra are reported as singlet (s), doublet (d), triplet (t), quartet (q), multiplet (m), broad (br), or a combination of these, to describe the observed spin–spin coupling pattern. Spin–spin coupling constants are reported in Hertz (Hz) and are uncorrected. Two-dimensional NMR spectroscopy (COSY, HSQC, HMBC, NOESY) was employed where appropriate to assist the assignment of signals in the ^1^H and ^13^C NMR spectra. Selected resonances were assigned to confirm connectivity. IR spectra were obtained on a Shimadzu FTIR-8400 instrument with a Golden Gate^TM^ attachment using a type IIa diamond as a single reflection element for the IR spectra of the solid or liquid compounds to be detected directly (thin layer). High-resolution mass spectra (HRMS) were recorded using ESI or EI conditions by the analytical services at the University of Glasgow. Liquid chromatography–mass spectrometry (LCMS) was recorded on a Thermo Scientific Dionex UltiMate 3000 LC system coupled with a Thermo Scientific LCQ Fleet quadrupole mass spectrometer using positive mode electrospray ionisation (ESI+). A Dr Maisch GmbH Reprosil Gold 120 C18 3 μm 150x4 mm column was used with UV absorption detected at 214 nm. A linear gradient of 5% to 95% HPLC-grade acetonitrile in ultra-pure water with 0.1% trifluoroacetic acid over 10 minutes was utilised with a flow rate of 1 mL min^–1^. Semi-preparative reverse-phase HPLC was performed on a Gilson HPLC system equipped with Gilson 306 pumps, a Phenomenex Synergi C18 (80 Å, 10 µm, 250 x 21.2 mm) column at a flow rate of 8 mL min^–1^. Non-linear gradients between 5% to 100% HPLC grade acetonitrile in ultra-pure water with 0.1% trifluoroacetic acid were utilised. UV absorption was detected at 214 nm and 280 nm using a Gilson 155 UV/VIS detector. Collected fractions were then lyophilised using a Thermo Heto PowerDry LL3000 lyophiliser.

VHL PROTAC 1 and epimer control 4:

**5**: To a stirred to solution of 4-bromo-2-hydroxybenzonitrile (9.0 g, 45 mmol, 1 equiv) in *N,N*-dimethylacetamide (153 mL, 0.3 m) was added 4-methylthiazole (8.3 mL, 91 mmol, 2 equiv), palladium(II) acetate (99 mg, 0.45 mmol, 1 mol%) and potassium acetate (9.0 g, 91 mmol, 2 equiv) and the resulting suspension was stirred at 150 °C for 16 hours. The reaction mixture was allowed to cool to room temperature, diluted with water (200 mL) and extracted with ethyl acetate (3 × 200 mL). The combined organic extracts were washed with brine (2 × 200 mL) and 5% aqueous lithium chloride (2 × 200 mL), dried over magnesium sulfate, filtered and concentrated *in vacuo.* Purification by column chromatography on silica gel using an eluent of 30% to 100% ethyl acetate in dichloromethane afforded nitrile **5** (4.7 g, 22 mmol, 53%) as a light yellow amorphous solid.

Analytical data observed were in accordance with literature values.^1^

^1^H NMR (400 MHz, methanol-*d_4_*) δ 8.95 (1H, s, thiazole-CH), 7.64 – 7.57 (1H, m, Ar-CH), 7.12 – 7.02 (2H, m, 2 × Ar-CH), 2.53 (3H, s, -CH_3_). ^13^C NMR (101 MHz, methanol-*d_4_*) δ 161.7 (Ar-C), 153.9 (thiazole-CH), 150.5 (thiazole-C), 139.3 (Ar-C), 134.9 (Ar-CH), 131.9 (thiazole-C), 121.6 (Ar-**C^a^**H), 117.4 (nitrile-C), 117.3 (Ar-CH), 100.4 (Ar-C), 16.2 (-CH_3_). LCMS (ESI) mass calculated for C_11_H_9_N_2_OS [M+H]^+^ m/z 217.04, found m/z 217.17 with *t*_R_ = 5.40 min.

**6**: To a dry flask charged with argon was added anhydrous tetrahydrofuran (6 mL, 0.2 m) and nitrile **5** (250 mg, 1.16 mmol, 1 equiv) and the reaction mixture was cooled to 0 °C. Lithium aluminium hydride (216 mg, 5.80 mmol, 5 equiv) was added portion-wise and the resulting suspension was stirred at 0 °C for 1 hour, then allowed to warm to room temperature and stirred for a further 16 hours. Once complete consumption of the starting material was confirmed by TLC, the reaction mixture was diluted with anhydrous tetrahydrofuran (10 mL) and cooled to 0 °C. The reaction was quenched by dropwise addition of water (216 μL), followed by 3 m aqueous sodium hydroxide (216 μL) and water (3 × 216 μL), and the resulting suspension was stirred vigorously at room temperature for 30 minutes. The precipitate was filtered and washed with a copious amount of tetrahydrofuran, and the filtrate was concentrated *in vacuo* to afford amine **6** in quantitative yield (255 mg, 1.16 mmol) as a dark yellow oil. The crude material was used in the next step without further purification.

Analytical data observed were in accordance with literature values.^1^

^1^H NMR (500 MHz, methanol-*d_4_*) δ 8.85 (1H, s, thiazole-CH), 7.31 (1H, d, *J* = 7.7 Hz, Ar-CH), 6.99 (1H, d, *J* = 1.7 Hz, Ar-CH), 6.92 (1H, dd, *J* = 7.7, 1.7 Hz, Ar-CH), 4.06 (2H, s, -CH_2_), 2.48 (3H, s, -CH_3_). ^13^C NMR (101 MHz, methanol-*d_4_*) δ 157.7 (Ar-C), 153.1 (thiazole-CH), 149.4 (thiazole-C), 135.3 (Ar-C), 132.2 (Ar-CH), 121.6 (Ar-CH), 116.9 (Ar-CH), 101.4 (Ar-C), 63.6 (thiazole-C), 53.7 (-CH_2_), 16.0 (-CH_3_). HRMS (ESI) exact mass calculated for C_11_H_13_N_2_O_6_S [M+H]^+^ m/z 221.0743, found m/z 221.0746. LCMS (ESI) mass calculated for C_11_H_13_N_2_O_6_S [M+H]^+^ m/z 221.07, found m/z 221.00 with *t*_R_ = 4.02 min.

**7a**: To a pre-stirred solution of *N*-Boc-*trans*-4-hydroxy-l-proline (525 mg, 2.27 mmol, 1 equiv), HATU (863 mg, 2.27 mmol, 1 equiv), OxymaPure (323 mg, 2.27 mmol, 1 equiv) and *N,N*-diisopropylethylamine (0.81 mL, 4.5 mmol, 2 equiv) in *N,N*-dimethylformamide (3.8 mL) was added amine **6** (500 mg, 2.27 mmol, 1 equiv) in *N,N*-dimethylformamide (3.8 mL, 0.3 m total concentration) and the resulting solution was stirred at room temperature for 16 hours. The reaction mixture was partitioned between dichloromethane (50 mL) and water (50 mL), and the aqueous phase was extracted with dichloromethane (5 × 50 mL). The combined organic phases were washed with 5% aqueous lithium chloride (5 × 100 mL), dried over magnesium sulfate, filtered and concentrated *in vacuo*. Purification by column chromatography on silica gel with an eluent of 5% to 30% ethanol in dichloromethane afforded *trans*-Hyp intermediate **7a** (533 mg, 1.21 mmol, 53%) as an orange amorphous solid.

Analytical data observed were in accordance with literature values.^1^

^1^H NMR (400 MHz, methanol-*d_4_*) δ 8.85 (1H, s, thiazole-CH), 7.29 (1H, d, *J* = 7.8 Hz, Ar-CH), 6.94 – 6.86 (2H, m, 2 × Ar-CH), 4.51 – 4.42 (1H, m, -C*H*OH), 4.41 – 4.36 (2H, m, -CH_2_), 4.35 – 4.27 (1H, m, -CH), 3.59 – 3.53 (1H, m, -C*H*H), 3.53 – 3.45 (1H, m, -CH*H*), 2.47 (3H, s, -CH_3_), 2.33 – 2.17 (1H, m, -C*H*H), 2.08 – 1.98 (1H, m, -CH*H*), 1.37 (9H, s, 3 × CH_3_). ^13^C NMR (101 MHz, methanol-*d_4_*) δ 176.1 (-CONH), 157.1 (Ar-C), 156.1 (-NCOOR), 152.7 (thiazole-CH), 148.9 (thiazole-C), 133.4 (Ar-C), 131.7 (Ar-CH), 126.2 (Ar-C), 121.5 (Ar-CH), 117.5 (Ar-CH), 81.7 (-*C*(CH_3_)_3_), 70.1 (-CHOH), 60.7 (thiazole-C), 58.3 (-CH), 56.0 (-CH_2_), 40.8 (-CH_2_), 39.7 (-CH_2_), 28.4 (3C, -C(*C*H_3_)_3_), 15.9 (-CH_3_). HRMS (ESI) exact mass calculated for C_21_H_27_N_3_O_5_SNa [M+Na]^+^ m/z 456.1564, found m/z 456.1570. LCMS (ESI) mass calculated for C_21_H_28_N_3_O_5_S [M+H]^+^ m/z 434.17, found m/z 434.17 with *t*_R_ = 4.96 min.

**7b**: To a pre-stirred solution of *N*-Boc-*cis*-4-hydroxy-l-proline (441 mg, 1.91 mmol, 1 equiv), HATU (723 mg, 1.91 mmol, 1 equiv), OxymaPure (271 mg, 1.91 mmol, 1 equiv) and *N,N*-diisopropylethylamine (0.68 mL, 3.8 mmol, 2 equiv) in *N,N*-dimethylformamide (3 mL) was added amine **6** (500 mg, 2.27 mmol, 1 equiv) in *N,N*-dimethylformamide (3 mL, 0.3 m total concentration) and the resulting solution was stirred at room temperature for 16 hours. The reaction mixture was partitioned between dichloromethane (50 mL) and water (50 mL), and the aqueous phase was extracted with dichloromethane (5 × 50 mL). The combined organic phases were washed with 5% aqueous lithium chloride (5 × 100 mL), dried over magnesium sulfate, filtered and concentrated *in vacuo*. Purification by column chromatography on silica gel with an eluent of 5% to 30% ethanol in dichloromethane afforded *cis*-Hyp intermediate **7b** (420 mg, 0.96 mmol, 50%) as an orange amorphous solid.

Analytical data observed were in accordance with literature values.^2^

^1^H NMR (400 MHz, methanol-*d_4_*) δ 8.85 (1H, s, thiazole-CH), 7.33 (1H, d, *J* = 7.6 Hz, Ar-CH), 6.95 – 6.85 (2H, m, 2 × Ar-CH), 4.51 – 4.42 (1H, m, -C*H*OH), 4.41 – 4.29 (2H, m, -CH_2_), 4.27 – 4.17 (1H, m, -CH), 3.59 – 3.52 (1H, m, -C*H*H), 3.49 – 3.41 (1H, m, -CH*H*), 2.48 (3H, s, -CH_3_), 2.45 – 2.35 (1H, m, -C*H*H), 2.06 – 1.96 (1H, m, -CH*H*), 1.38 (9H, s, 3 × CH_3_). ^13^C NMR (101 MHz, methanol-*d_4_*) δ 176.1 (-CONH), 157.1 (Ar-C), 156.1 (-NCOOR), 152.8 (thiazole-CH), 148.9 (thiazole-C), 133.3 (Ar-C), 131.6 (Ar-CH), 126.1 (Ar-C), 121.4 (Ar-CH), 117.2 (Ar-CH), 81.9 (-*C*(CH_3_)_3_), 70.4 (-CHOH), 61.5 (thiazole-C), 61.1 (-CH), 56.0 (-CH_2_), 39.7 (-CH_2_), 28.7 (-CH_2_), 28.4 (3C, -C(*C*H_3_)_3_), 15.9 (-CH_3_). HRMS (ESI) exact mass calculated for C_21_H_27_N_3_O_5_S [M-H]^-^ m/z 433.1683, found m/z 433.1612.

**8a**: Protected *trans*-Hyp intermediate **7a** (140 mg, 0.32 mmol, 1 equiv) was dissolved in trifluoroacetic acid/dichloromethane (5 mL/5 mL, 0.03 m) and the resulting solution was stirred at room temperature for 2 hours. The reaction was monitored by TLC for complete consumption of the starting material before removal of volatile components *in vacuo*. To the residue was added to a pre-stirred solution of *N*-Boc-l*-tert*-leucine (75 mg, 0.32 mmol, 1 equiv), HATU (120 mg, 0.32 mmol, 1 equiv), OxymaPure (46 mg, 0.32 mmol, 1 equiv) and *N,N*-diisopropylethylamine (0.23 mL, 1.3 mmol, 4 equiv) in *N,N*-dimethylformamide (1 mL, 0.3 m), and the resulting solution was stirred at room temperature for 16 hours. The reaction mixture was partitioned between dichloromethane (20 mL) and water (20 mL), and the aqueous phase was extracted with dichloromethane (3 × 20 mL). The combined organic phases were washed with 5% aqueous lithium chloride (3 × 20 mL), dried over magnesium sulfate, filtered and concentrated *in vacuo*. Purification by column chromatography on silica gel with an eluent of 0% to 20% ethanol in ethyl acetate afforded *tert*-leucine intermediate **8a** (110 mg, 0.20 mmol, 60%) as a yellow oily solid.

Analytical data observed were in accordance with literature values.^1^

^1^H NMR (400 MHz, methanol-*d_4_*) δ 8.85 (1H, s, thiazole-CH), 7.98 (1H, s, -NHCOOR), 7.36 (1H, d, *J* = 8.2 Hz, Ar-CH), 6.94 – 6.85 (2H, m, 2 × Ar-CH), 4.63 – 4.56 (1H, m, -C*H*OH), 4.50 (1H, s, -CH), 4.44 – 4.34 (2H, m, -CH_2_), 4.32 – 4.24 (1H, m, -CH), 3.90 – 3.76 (2H, m, -CH_2_), 2.48 (3H, s, -CH_3_), 2.24 – 2.06 (2H, m, -CH_2_), 1.44 (9H, s, 3 × CH_3_), 1.00 (9H, s, 3 × CH_3_). ^13^C NMR (101 MHz, methanol-*d_4_*) δ 174.5 (-CONH), 172.9 (-CONH), 167.4 (-NHCOOR), 156.6 (Ar-C), 152.6 (thiazole-CH), 148.8 (thiazole-C), 133.5 (Ar-C), 132.7 (Ar-CH), 126.2 (Ar-C), 121.4 (Ar-CH), 116.8 (Ar-CH), 80.6 (-*C*(CH_3_)_3_), 71.0 (-CHOH), 60.6 (thiazole-C), 60.4 (-CH), 57.9 (-CH), 56.0 (-CH_2_), 39.5 (-CH_2_), 38.9 (-CH_2_), 36.8 (-*C*(CH_3_)_3_), 28.7 (3C, -C(*C*H_3_)_3_), 26.9 (3C, -C(*C*H_3_)_3_), 16.0 (-CH_3_). HRMS (ESI) exact mass calculated for C_27_H_39_N_4_O_6_S [M+H]^+^ m/z 547.2585, found m/z 547.2580. LCMS (ESI) mass calculated for C_27_H_39_N_4_O_6_S [M+H]^+^ m/z 547.26, found m/z 547.08 with *t*_R_ = 5.50 min.

**8b**: Protected *cis*-Hyp intermediate **7b** (410 mg, 0.95 mmol, 1 equiv) was dissolved in trifluoroacetic acid/dichloromethane (10 mL/10 mL, 0.03 m) and the resulting solution was stirred at room temperature for 2 hours. The reaction was monitored by TLC for complete consumption of the starting material before removal of volatile components *in vacuo*. To the residue was added a pre-stirred solution of *N*-Boc-l*-tert*-leucine (220 mg, 0.95 mmol, 1 equiv), HATU (360 mg, 0.95 mmol, 1 equiv), OxymaPure (130 mg, 0.95 mmol, 1 equiv) and *N,N*-diisopropylethylamine (0.67 mL, 3.8 mmol, 4 equiv) in *N,N*-dimethylformamide (3.2 mL, 0.3 m), and the resulting solution was stirred at room temperature for 16 hours. The reaction mixture was partitioned between dichloromethane (30 mL) and water (30 mL), and the aqueous phase was extracted with dichloromethane (3 × 30 mL). The combined organic phases were washed with 5% aqueous lithium chloride (3 × 40 mL), dried over magnesium sulfate, filtered and concentrated *in vacuo*. Purification by column chromatography on silica gel with an eluent of 0% to 20% ethanol in ethyl acetate afforded the *tert*-leucine intermediate **8b** (260 mg, 0.47 mmol, 50%) as a yellow oily solid.

Analytical data observed were in accordance with literature values.^2^

^1^H NMR (400 MHz, MHz, methanol-*d_4_*) δ 8.85 (1H, s, thiazole-CH), 7.98 (1H, s, -NHCOOR), 7.35 (1H, d, *J* = 7.6 Hz, Ar-CH), 6.93 – 6.83 (2H, m, 2 × Ar-CH), 4.58 – 4.51 (1H, m, -C*H*OH), 4.47 – 4.35 (3H, -CH_2_ & -CH), 4.25 – 4.18 (1H, m, -CH), 4.03 – 3.95 (1H, m, -C*H*H), 3.73 – 3.66 (1H, m, -CH*H*), 2.48 (3H, s, -CH_3_), 2.46 – 2.38 (1H, m, -C*H*H), 2.01 – 1.94 (1H, m, -CH), 1.43 (9H, s, 3 × CH_3_), 0.99 (9H, s, 3 × CH_3_). ^13^C NMR (101 MHz, methanol-*d_4_*) δ 175.0 (-CONH), 173.3 (-CONH), 168.6 (-NHCOOR), 156.7 (Ar-C), 152.7 (thiazole-CH), 148.8 (thiazole-C), 133.6 (Ar-C), 132.8 (Ar-CH), 125.9 (Ar-C), 121.4 (Ar-CH), 116.6 (Ar-CH), 80.7 (-*C*(CH_3_)_3_), 71.5 (-CHOH), 61.5 (thiazole-C), 61.0 (-CH), 57.7 (-CH), 54.8 (-CH_2_), 39.6 (-CH_2_), 38.9 (-CH_2_), 36.2 (-*C*(CH_3_)_3_), 28.7 (3C, -C(*C*H_3_)_3_), 26.9 (3C, -C(*C*H_3_)_3_), 15.9 (-CH_3_). HRMS (ESI) exact mass calculated for C_27_H_39_N_4_O_6_S [M+H]^+^ m/z 547.2585, found m/z 547.2579.

**9a**: Protected *tert*-leucine intermediate **8a** (100 mg, 0.18 mmol, 1 equiv) was dissolved in trifluoroacetic acid/dichloromethane (3 mL/3 mL, 0.03 m) and the resulting solution was stirred at room temperature for 2 hours. The reaction was monitored by TLC for complete consumption of the starting material before removal of volatile components *in vacuo*. To the residue was added a pre-stirred solution of 1-fluorocyclopropanecarboxylic acid (19 mg, 0.18 mmol, 1 equiv), HATU (70 mg, 0.18 mmol, 1 equiv), OxymaPure (26 mg, 0.18 mmol, 1 equiv) and *N,N*-diisopropylethylamine (0.13 mL, 0.73 mmol, 4 equiv) in *N,N*-dimethylformamide (0.6 mL, 0.3 m), and the resulting solution was stirred at room temperature for 16 hours. The reaction mixture was partitioned between dichloromethane (15 mL) and water (15 mL), and the aqueous phase was extracted with dichloromethane (3 × 15 mL). The combined organic phases were washed with 5% aqueous lithium chloride (3 × 20 mL), dried over magnesium sulfate, filtered and concentrated *in vacuo*. Purification by column chromatography on silica gel with an eluent of 0% to 10% ethanol in ethyl acetate afforded active VHL ligand **9a** (72 mg, 0.14 mmol, 74%) as a pale yellow amorphous solid.

Analytical data observed were in accordance with literature values.^1^

^1^H NMR (500 MHz, chloroform-*d*) δ 9.30 (1H, s, phenol-OH), 8.64 (1H, s, thiazole-CH), 8.09 – 8.03 (1H, m, -CONH), 7.09 (1H, d, *J* = 7.8 Hz, Ar-CH), 7.05 – 7.00 (1H, m, -CONH), 6.94 (1H, d, *J* = 1.8 Hz, Ar-CH), 6.83 (1H, dd, *J* = 7.8, 1.8 Hz, Ar-CH), 4.67 (1H, t, *J* = 7.9 Hz, -CH), 4.51 – 4.45 (2H, m, -CH & -C*H*OH), 4.42 – 4.35 (1H, m, -C*H*H), 4.20 – 4.14 (1H, m, -CH*H*), 4.02 (1H, br s, hydroxyproline-OH), 3.96 – 3.88 (1H, m, -C*H*H), 3.65 – 3.58 (1H, m, -CH*H*), 2.48 (3H, s, -CH_3_), 2.40 – 2.33 (1H, m, -C*H*H), 2.08 – 2.01 (1H, m, -CH*H*), 1.34 – 1.17 (4H, m, 2 × cyclopropyl-CH_2_), 0.90 (9H, s, 3 × CH_3_). ^13^C NMR (126 MHz, chloroform-*d*) δ 172.9 (-CONH), 171.1 (-CONH), 170.4 (d, *^2^J_C-F_* = 20.2 Hz, -CONH), 155.8 (Ar-C), 150.5 (thiazole-CH), 148.4 (thiazole-C), 133.3 (thiazole-C), 131.7 (Ar-C), 131.2 (Ar-CH), 124.1 (Ar-C), 120.9 (Ar-CH), 118.2 (Ar-CH), 78.3 (d, *^1^J_C-F_* = 229.7 Hz, -CF), 70.2 (-CHOH), 58.5 (-CH), 57.8 (-CH), 56.9 (-CH_2_), 40.0 (-CH_2_), 36.2 (-*C*(CH_3_)_3_), 35.7 (-CH_2_), 26.3 (3C, -C(*C*H_3_)_3_), 16.2 (-CH_3_), 13.9 (d, *^2^J_C-F_* = 10.2 Hz, cyclopropyl-CH_2_), 13.7 (d, *^2^J_C-F_* = 10.1 Hz, cyclopropyl-C^a^H_2_). HRMS (ESI) exact mass calculated for C_26_H_33_FN_4_O_5_SNa [M+Na]^+^ m/z 555.2053, found m/z 555.2058. LCMS (ESI) mass calculated for C_26_H_34_FN_4_O_5_S [M+H]^+^ m/z 533.22, found m/z 533.25 with *t*_R_ = 5.25 min.

**9b**: Protected *tert*-leucine intermediate **8b** (120 mg, 0.22 mmol, 1 equiv) was dissolved in trifluoroacetic acid/dichloromethane (3.8 mL/3.8 mL, 0.03 m) and the resulting solution was stirred at room temperature for 2 hours. The reaction was monitored by TLC for complete consumption of the starting material before removal of volatile components *in vacuo*. To the residue was added a pre-stirred solution of 1-fluorocyclopropanecarboxylic acid (23 mg, 0.22 mmol, 1 equiv), HATU (84 mg, 0.22 mmol, 1 equiv), OxymaPure (31 mg, 0.22 mmol, 1 equiv) and *N,N*-diisopropylethylamine (0.16 mL, 0.88 mmol, 4 equiv) in *N,N*-dimethylformamide (0.7 mL, 0.3 m), and the resulting solution was stirred at room temperature for 16 hours. The reaction mixture was partitioned between dichloromethane (15 mL) and water (15 mL), and the aqueous phase was extracted with dichloromethane (3 × 15 mL). The combined organic phases were washed with 5% aqueous lithium chloride (3 × 20 mL), dried over magnesium sulfate, filtered and concentrated *in vacuo*. Purification by column chromatography on silica gel with an eluent of 0% to 10% ethanol in ethyl acetate afforded epimer **9b** (73 mg, 0.14 mmol, 62%) as a pale yellow amorphous solid.

Analytical data observed were in accordance with literature values.^2^

^1^H NMR (500 MHz, chloroform-*d*) δ 8.80 – 8.63 (2H, m, phenol-OH & thiazole-CH), 8.11 – 8.04 (1H, m, -CONH), 7.15 (1H, d, *J* = 7.8 Hz, Ar-CH), 6.98 (1H, d, *J* = 1.8 Hz, Ar-CH), 6.90 (1H, dd, *J* = 7.8, 1.8 Hz, Ar-CH), 6.89 – 6.86 (1H, m, -CONH), 5.15 (1H, s, hydroxyproline-OH), 4.77 (1H, d, *J* = 9.0 Hz, -CH), 4.60 – 4.51 (1H, m, -C*H*H), 4.51 – 4.43 (2H, m, -CH & -CHOH), 4.19 (1H, dd, *J* = 14.7, 5.2 Hz, -CH*H*), 3.87 (1H, dd, *J* = 11.0, 4.3 Hz, -C*H*H), 3.78 (1H, dd, *J* = 11.0, 1.3 Hz, -CH*H*), 2.52 (3H, s, -CH_3_), 2.35 (1H, d, *J* = 14.3 Hz, -C*H*H), 2.20 (1H, ddd, *J* = 14.3, 9.2, 5.0 Hz, -CH*H*), 1.36 – 1.20 (4H, m, 2 × cyclopropyl-CH_2_), 0.84 (9H, s, 3 × CH_3_). ^13^C NMR (126 MHz, chloroform-*d*) δ 173.9 (-CONH), 171.9 (-CONH), 170.0 (d, *^2^J_C-F_* = 20.3 Hz, -CONH), 155.6 (Ar-C), 150.6 (thiazole-CH), 148.6 (thiazole-C), 133.8 (thiazole-C), 131.6 (Ar-C), 131.2 (Ar-CH), 123.3 (Ar-C), 121.3 (Ar-CH), 118.6 (Ar-CH), 78.0 (d, *^1^J_C-F_* = 241.0 Hz, -C^b^F), 71.2 (-CHOH), 59.6 (-CH), 58.7 (-CH), 57.2 (-CH_2_), 40.1 (-CH_2_), 35.3 (-*C*(CH_3_)_3_), 34.9 (-CH_2_), 26.2 (3C, -C(*C*H_3_)_3_), 16.3 (-CH_3_), 13.8 (d, *^2^J_C-F_* = 10.2 Hz, cyclopropyl-CH_2_), 13.7 (d, *^2^J_C-F_* = 10.2 Hz, cyclopropyl-CH_2_). HRMS (ESI) exact mass calculated for C_26_H_33_FN_4_O_5_SNa [M+Na]^+^ m/z 555.2053, found m/z 555.2052. LCMS (ESI) mass calculated for C_26_H_34_FN_4_O_5_S [M+H]^+^ m/z 533.22, found m/z 533.17 with *t*_R_ = 5.28 min.

**10**: To a dry flask charged with argon was added anhydrous tetrahydrofuran/*N,N*-dimethylformamide (1.5 mL/1.5 mL, 1 m), pentaethylene glycol (1.5 mL, 7.0 mmol, 5 equiv) and sodium hydride (60% in mineral oil, 140 mg, 2.5 mmol, 2.5 equiv) at 0 °C. The resulting suspension was stirred for 30 minutes before 1-chloro-6-iodohexane (0.21 mL, 1.7 mmol, 1 equiv) was added slowly at 0 °C. The reaction mixture was allowed to reach room temperature and stirred for further 16 hours. The reaction was quenched by dropwise addition of water (10 mL) before addition of 1 m aqueous hydrochloric acid (10 mL). The aqueous phase was extracted with chloroform (3 × 20 mL) and the combined organic extracts were washed with 5% aqueous lithium chloride (3 × 40 mL), dried over magnesium sulfate, filtered and concentrated *in vacuo*. Purification by column chromatography on silica gel with an eluent of 75% to 100% ethyl acetate in chloroform afforded alcohol **10** (380 mg, 1.06 mmol, 79%) as a clear oil.

Analytical data observed were in accordance with literature values.^1^

^1^H NMR (400 MHz, chloroform-*d*) δ 3.76 – 3.70 (2H, m, -CH_2_), 3.69 – 3.57 (18H, m, 9 × -CH_2_), 3.53 (2H, t, *J* = 6.7 Hz, -CH_2_), 3.45 (2H, t, *J* = 6.6 Hz, -CH_2_), 2.55 (1H, t, *J* = 6.3 Hz, -OH), 1.82 – 1.73 (2H, m, -CH_2_), 1.63 – 1.54 (2H, m, -CH_2_), 1.50 – 1.32 (4H, m, 2 × -CH_2_). ^13^C NMR (101 MHz, chloroform-*d*) δ 72.7 (-CH_2_), 71.4 (-CH_2_), 70.8 (-CH_2_), 70.8 (-CH_2_), 70.7 (2 × -CH_2_), 70.7 (-CH_2_), 70.7 (-CH_2_), 70.5 (-CH_2_), 70.2 (-CH_2_), 61.9 (-CH_2_OH), 45.2 (-CH_2_Cl), 32.7 (-CH_2_), 29.6 (-CH_2_), 26.9 (-CH_2_), 25.6 (-CH_2_). HRMS (ESI) exact mass calculated for C_16_H_33_ClO_6_Na [M+Na]^+^ m/z 379.1858, found m/z 379.1847. LCMS (ESI) mass calculated for C_16_H_34_ClO_6_ [M+H]^+^ m/z 357.20, found m/z 357.17 with *t*_R_ = 5.81 min.

**11**: To a dry flask charged with argon was added anhydrous dichloromethane (19 mL, 0.1 m), alcohol **10** (662 mg, 1.86 mmol, 1 equiv), methanesulfonyl chloride (0.22 mL, 2.8 mmol, 1.5 equiv) and triethylamine (0.76 mL, 5.6 mmol, 3 equiv) and the resulting solution was stirred at room temperature for 16 hours. The reaction mixture was partitioned between 1 m aqueous hydrochloric acid (20 mL) and dichloromethane (20 mL), and the aqueous phase was extracted with dichloromethane (3 × 20 mL). The combined organic extracts were washed with water (2 × 40 mL), dried over magnesium sulfate, filtered and concentrated *in vacuo*. Purification by column chromatography on silica gel with ethyl acetate afforded mesylate **11** (662 mg, 1.40 mmol, 75%) as a light yellow oil.

^1^H NMR (400 MHz, chloroform-*d*) δ 4.43 – 4.34 (2H, m, -CH_2_), 3.80 – 3.73 (2H, m, -CH_2_), 3.71 – 3.60 (16H, m, 8 × -CH_2_), 3.60 – 3.49 (2H, m, -CH_2_), 3.45 (2H, t, *J* = 6.6 Hz, -CH_2_), 3.08 (3H, s, -CH_3_), 1.83 – 1.72 (2H, m, -CH_2_), 1.66 – 1.54 (2H, m, -CH_2_), 1.52 – 1.30 (4H, m, 2 × -CH_2_). ^13^C NMR (101 MHz, chloroform-*d*) δ 71.4 (-CH_2_), 70.8 (-CH_2_), 70.8 (-CH_2_), 70.8 (2 × -CH_2_), 70.8 (2 × -CH_2_), 70.7 (-CH_2_), 70.3 (-CH_2_), 69.4 (-CH_2_), 69.2 (-CH_2_), 45.2 (-CH_2_Cl), 37.9 (-CH_3_), 32.7 (-CH_2_), 29.6 (-CH_2_), 26.9 (-CH_2_), 25.6 (-CH_2_). IR (thin film) 1349, 1173 cm^-1^. HRMS (ESI) exact mass calculated for C_17_H_35_O_8_SClNa [M+Na]^+^ m/z 457.1633, found m/z 457.1633.

**VHL HaloPROTAC 1**: To a stirred solution of mesylate **11** (89 mg, 0.21 mmol, 1.5 equiv) in *N,N*-dimethylformamide (0.35 mL, 0.4 m) was added phenol **9a** (73 mg, 0.14 mmol, 1 equiv) and potassium carbonate (48 mg, 0.35 mmol, 2.5 equiv), the resulting solution was heated to 70 °C and stirred for 16 hours. The reaction mixture was diluted with water (15 mL) and the aqueous phase was extracted with dichloromethane (4 × 15 mL). The combined organic extracts were washed with 5% aqueous lithium chloride (3 × 20 mL), dried over magnesium sulfate, filtered and concentrated *in vacuo*. Purification by column chromatography on silica gel with an eluent of 5% to 20% ethanol in dichloromethane, followed by reverse phase HPLC using a focused gradient across 50% and 80% acetonitrile in water over 30 minutes eluted the title compound at *t*_R_ = 23 minutes (76% acetonitrile). Lyophilisation of the product fraction afforded **VHL HaloPROTAC 1** (30 mg, 0.034 mmol, 25%) as a white fluffy powder.

^1^H NMR (400 MHz, methanol-*d_4_*) δ 9.97 (1H, s, thiazole-CH), 7.61 (1H, d, *J* = 7.8 Hz, Ar-CH), 7.17 (1H, d, *J* = 1.6 Hz, Ar-CH), 7.12 (1H, dd, *J* = 7.8, 1.4 Hz, Ar-CH), 4.75 (1H, s, -CH), 4.67 – 4.59 (1H, m, -CH), 4.58 – 4.48 (2H, m, -CH & -C*H*H), 4.45 – 4.35 (2H, m, -CH*H*), 4.33 – 4.23 (2H, m, -CH_2_), 3.95 – 3.90 (2H, m, -CH_2_), 3.89 – 3.83 (1H, m, -C*H*H), 3.83 – 3.77 (1H, m, -CH*H*), 3.77 – 3.72 (2H, m, -CH_2_), 3.71 – 3.66 (2H, m, -CH_2_), 3.65 – 3.59 (10H, m, 5 × -CH_2_), 3.58 – 3.52 (4H, m, 2 × -CH_2_), 3.46 (2H, t, *J* = 6.5 Hz, -CH_2_), 2.62 (3H, s, -CH_3_), 2.30 – 2.22 (1H, m, -C*H*H), 2.14 – 2.04 (1H, m, -CH*H*), 1.80 – 1.70 (2H, m, -CH_2_), 1.61 – 1.52 (2H, m, -CH_2_), 1.49 – 1.26 (8H, m, 4 × -CH_2_), 1.03 (9H, s, 3 × CH_3_). ^13^C NMR (101 MHz, methanol-*d_4_*) δ 174.4 (-CONH), 171.7 (-CONH), 171.4 (d, *^2^J_C-F_* = 20.6 Hz, -CONH), 158.4 (Ar-C), 156.5 (thiazole-CH), 142.5 (thiazole-C), 137.6 (thiazole-C), 130.7 (Ar-C), 130.3 (Ar-CH), 129.1 (Ar-C), 122.8 (Ar-CH), 113.7 (Ar-CH), 79.2 (d, *^1^J_C-F_* = 231.4 Hz, -CF), 72.1 (-CH_2_), 71.8 (-CH_2_), 71.6 (2 × -CH_2_), 71.6 (2 × -CH_2_), 71.5 (2 × -CH_2_), 71.2 (-C), 71.1 (-CH_2_), 70.8 (-CH_2_), 69.6 (-CH_2_), 60.8 (-CH), 58.7 (-CH), 58.2 (-CH_2_), 45.7 (-CH_2_), 39.4 (-CH_2_), 39.0 (-CH_2_), 37.3 (-*C*(CH_3_)_3_), 33.7 (-CH_2_), 30.5 (-CH_2_), 27.7 (-CH_2_), 26.9 (3 × -CH_3_), 26.5 (-CH_2_), 14.0 (d, *^2^J_C-F_* = 11.0 Hz, cyclopropyl-CH_2_), 13.9 (d, *^2^J_C-F_* = 10.9 Hz, cyclopropyl-CH_2_), 13.2 (-CH_3_). IR (thin film) 3320, 1094, 668 cm^-1^. HRMS (ESI) exact mass calculated for C_42_H_65_ClFN_4_O_10_S [M+H]^+^ m/z 871.4088, found m/z 871.4101. LCMS (ESI) mass calculated for C_42_H_65_ClFN_4_O_10_S [M+H]^+^ m/z 871.41, found m/z 871.75 with *t*_R_ = 6.35 min.

**Epimer control 4**: To a stirred solution of mesylate **11** (27 mg, 0.062 mmol, 1.5 equiv) in *N,N*-dimethylformamide (0.11 mL, 0.4 m) was added phenol **9b** (22 mg, 0.041 mmol, 1 equiv) and potassium carbonate (15 mg, 0.10 mmol, 2.5 equiv), the resulting solution was heated to 70 °C and stirred for 16 hours. Complete consumption of the starting material was confirmed by TLC before removing volatile components *in vacuo*. Purification by column chromatography on silica gel with an eluent of 5% to 20% ethanol in dichloromethane, followed by reverse phase HPLC using a focused gradient across 60% and 90% acetonitrile in water over 25 minutes eluted the title compound at *t*_R_ = 19 minutes (78% acetonitrile). Lyophilisation of the product fraction afforded **epimer control 4** (15 mg, 0.017 mmol, 42%) as a white fluffy powder.

^1^H NMR (400 MHz, methanol-*d_4_*) δ 9.18 (1H, s, thiazole-CH), 7.49 (1H, d, *J* = 7.8 Hz, Ar-CH), 7.09 (1H, d, *J* = 1.6 Hz, Ar-CH), 7.04 (1H, dd, *J* = 7.7, 1.7 Hz, Ar-CH), 4.71 – 4.65 (1H, s, -CH), 4.61 – 4.52 (1H, m, -CH), 4.52 – 4.43 (1H, m, -CH), 4.44 – 4.35 (2H, m, -CH_2_), 4.31 – 4.21 (2H, m, -CH_2_), 3.95 – 3.88 (2H, m, -CH_2_), 3.78 – 3.51 (18H, m, 9 × -CH_2_), 3.49 – 3.42 (2H, m, -CH_2_), 2.53 (3H, s, -CH_3_), 2.50 – 2.39 (1H, m, -C*H*H), 2.03 – 1.92 (1H, m, -CH*H*), 1.82 – 1.70 (2H, m, -CH_2_), 1.65 – 1.23 (12H, m, 6 × -CH_2_), 1.02 (9H, s, 3 × CH_3_). ^13^C NMR (101 MHz, methanol-*d_4_*) δ 174.7 (-CONH), 171.9 (-CONH), 171.6 (d, *^2^J_C-F_* = 20.7 Hz, -CONH), 158.2 (Ar-C), 153.8 (thiazole-CH), 147.4 (thiazole-C), 134.6 (thiazole-C), 132.0 (Ar-C), 130.3 (Ar-CH), 128.8 (Ar-C), 122.8 (Ar-CH), 113.8 (Ar-CH), 79.1 (d, *^1^J_C-F_* = 231.7 Hz, -CF), 72.1 (-CH_2_), 71.8 (-CH_2_), 71.6 (-CH_2_), 71.6 (2 × -CH_2_), 71.6 (2 × -CH_2_), 71.5 (-CH_2_), 71.2 (-C), 71.0 (-CH_2_), 70.8 (-CH_2_), 69.5 (-CH_2_), 61.1 (-CH), 58.7 (-CH), 57.8 (-CH_2_), 45.7 (-CH_2_), 39.6 (-CH_2_), 37.8 (-CH_2_), 36.8 (-*C*(CH_3_)_3_), 33.8 (-CH_2_), 30.6 (-CH_2_), 27.7 (-CH_2_), 26.9 (3 × -CH_3_), 26.5 (-CH_2_), 15.2 (-CH_3_), 14.1 (d, *^2^J_C-F_* = 11.0 Hz, cyclopropyl-CH_2_), 13.9 (d, *^2^J_C-F_* = 10.9 Hz, cyclopropyl-CH_2_). HRMS (ESI) exact mass calculated for C_42_H_64_ClFN_4_O_10_SNa [M+Na]^+^ m/z 893.3908, found m/z 893.3873. LCMS (ESI) mass calculated for C_42_H_65_ClFN_4_O_10_S [M+H]^+^ m/z 871.41, found m/z 871.67 with *t*_R_ = 6.38 min.

VHL PROTAC 3:

**12**: To a stirred solution of alcohol **10** (330 mg, 0.93 mmol, 1 equiv) in dichloromethane (9.3 mL, 0.1 m) was added dimethyl sulfoxide (2.2 mL) and triethylamine (0.52 mL, 3.7 mmol, 4 equiv) and reaction mixture was cooled to 0 °C. To anhydrous dimethyl sulfoxide (2.2 mL) was added sulfur trioxide pyridine complex (370 mg, 2.3 mmol, 2.5 equiv) at 0 °C and stirred for 10 minutes before dropwise addition of the pre-stirred solution to the reaction mixture. The resulting solution was stirred for 1 hour at 0 °C and was then allowed to reach room temperature over 2 hours before complete consumption of the starting material was confirmed by LCMS. The reaction mixture was diluted with water (20 mL) and the aqueous phase was extracted with dichloromethane (3 × 20 mL). The combined organic extracts were washed with water (30 mL) and brine (30 mL), dried over magnesium sulfate, filtered and concentrated *in vacuo*. Aldehyde **12** (300 mg, 0.85 mmol, 91%) was afforded as a yellow oil and the crude material was used in the next step without further purification.

LCMS (ESI) mass calculated for C_16_H_32_ClO_6_ [M+H]^+^ m/z 355.19, found m/z 355.08 with *t*_R_ = 5.64 min.

**13**: To a stirred solution of aldehyde **12** (300 mg, 0.85 mmol, 1 equiv) in *tert*-butanol/water (7.2 mL/1.8 mL, 0.1 m) was added sodium phosphate monobasic monohydrate (224 mg, 1.44 mmol, 1.7 equiv), sodium chlorite (268 mg, 2.98 mmol, 3.5 equiv) and amylene (2 m in THF, 3.2 mL, 6.0 mmol, 7 equiv). The resulting solution was stirred at room temperature for 16 hours. The reaction mixture was diluted with water (10 mL) and brine (10 mL), and the aqueous phase was extracted with ethyl acetate (4 × 20 mL). The combined organic extracts were washed with brine (2 × 40 mL), dried over magnesium sulfate and concentrated *in vacuo*. Purification by column chromatography on silica gel with an eluent of 5% to 20% ethanol in dichloromethane yielded carboxylic acid **13** (260 mg, 0.70 mmol, 82%) as a clear oil.

Analytical data observed were in accordance with literature values.^1^

^1^H NMR (400 MHz, chloroform-*d*) δ 3.83 (2H, s, -CH_2_), 3.72 – 3.54 (17H, m, 8 × -CH_2_ & -COOH), 3.52 (2H, t, *J* = 6.7 Hz, -CH_2_), 3.43 (2H, t, *J* = 6.7 Hz, -CH_2_), 1.81 – 1.72 (2H, m, -CH_2_), 1.62 – 1.53 (2H, m, -CH_2_), 1.49 – 1.31 (4H, m, 2 × -CH_2_). ^13^C NMR (101 MHz, chloroform-*d*) δ 176.1 (-COOH), 71.3 (-CH_2_), 71.1 (-CH_2_), 70.5 (-CH_2_), 70.4 (-CH_2_), 70.2 (2 × -CH_2_), 70.1 (-CH_2_), 70.0 (-CH_2_), 69.9 (-CH_2_), 69.2 (-CH_2_), 45.2 (-CH_2_Cl), 32.7 (-CH_2_), 29.5 (-CH_2_), 26.8 (-CH_2_), 25.5 (-CH_2_). HRMS (ESI) exact mass calculated for C_16_H_31_ClO_7_Na [M+Na]^+^ m/z 393.1651, found m/z 393.1653. LCMS (ESI) mass calculated for C_16_H_32_ClO_7_ [M+H]^+^ m/z 371.18, found m/z 371.18 with *t*_R_ = 5.80 min.

**VHL HaloPROTAC 3**: To a pre-stirred solution of carboxylic acid **13** (49 mg, 0.13 mmol, 1 equiv), HATU (50 mg, 0.13 mmol, 1 equiv) and *N,N*-diisopropylethylamine (70 μL, 0.39 mmol, 3 equiv) in *N,N*-dimethylformamide (0.5 mL, 0.3 m) was added commercially obtained VH032 (57 mg, 0.13 mmol, 1 equiv), and the resulting solution was stirred at room temperature for 16 hours. The reaction mixture was partitioned between dichloromethane (15 mL) and water (15 mL), and the aqueous phase was extracted with dichloromethane (5 × 15 mL). The combined organic phases were washed with 5% aqueous lithium chloride (5 × 20 mL), dried over magnesium sulfate, filtered and concentrated *in vacuo*. Purification by column chromatography on silica gel with an eluent of 5% to 20% ethanol in dichloromethane, followed by reverse phase HPLC using a focussed gradient across 45% and 70% acetonitrile in water over 35 minutes eluted the title compound at *t*_R_ = 22 minutes (63% acetonitrile). Lyophilisation of the product fraction afforded **VHL HaloPROTAC 3** (36 mg, 0.050 mmol, 35%) as a white fluffy powder.

Analytical data observed were in accordance with literature values.^1^

^1^H NMR (500 MHz, methanol-*d_4_*) δ 9.08 (1H, s, thiazole-CH), 7.51 – 7.43 (4H, m, 4 × Ar-CH), 4.70 (1H, s, -CH), 4.60 – 4.47 (3H, m, -CHOH & -CH_2_), 4.37 (1H, d, *J* = 15.6 Hz, -CH), 4.04 (2H, m, -CH_2_), 3.88 (1H, dd, *J* = 11.2, 1.7 Hz, -C*H*H), 3.80 (1H, dd, *J* = 11.0, 3.8 Hz, -CH*H*), 3.73 – 3.52 (18H, m, 9 × -CH_2_), 3.46 (2H, t, *J* = 6.5 Hz, -CH_2_), 2.50 (3H, s, -CH_3_), 2.23 (1H, ddd, *J* = 13.2, 7.6, 1.9 Hz, -C*H*H), 2.09 (1H, ddd, *J* = 13.3, 9.1, 4.4 Hz, -CH*H*), 1.79 – 1.72 (2H, m, -CH_2_), 1.61 – 1.53 (2H, m, -CH_2_), 1.49 – 1.34 (4H, m, 2 × -CH_3_), 1.05 (9H, s, 3 × -CH_3_). ^13^C NMR (126 MHz, methanol-*d_4_*) δ 174.4 (-CONH), 172.1 (-CONH), 171.6 (-CONH), 153.5 (thiazole-CH), 147.9 (thiazole-C), 140.7 (Ar-C), 130.9 (thiazole-C), 130.4 (2 × Ar-CH), 129.6 (Ar-C), 129.0 (2 × Ar-CH), 72.3 (-CH_2_), 72.1 (-CH_2_), 71.7 (-CH_2_), 71.6 (-CH_2_), 71.6 (3 × -CH_2_), 71.5 (-CH_2_), 71.2 (-CH_2_), 71.1 (-CH_2_), 71.0 (-CHOH), 60.8 (-CH), 58.1 (-CH), 58.1 (-CH_2_), 45.7 (-CH_2_), 43.7 (-CH_2_), 38.9 (-CH_2_), 37.1 (-*C*(CH_3_)_3_), 33.7 (-CH_2_), 30.6 (-CH_2_), 27.7 (-CH_2_), 27.0 (3C, -C(*C*H_3_)_3_), 26.5 (-CH_2_), 15.4 (-CH_3_). HRMS (ESI) exact mass calculated for C_38_H_59_N_4_O_9_SClNa [M+Na]^+^ m/z 805.3583, found m/z 805.3554. LCMS (ESI) mass calculated for C_38_H_60_N_4_O_9_SCl [M+H]^+^ m/z 783.38, found m/z 783.08 with *t*_R_ = 6.16 min.

CRBN PROTAC 2:

**14**: To a stirred solution of 3-fluorophthalic anhydride (3.93 g, 23.7 mmol, 1 equiv) in anhydrous *N,N*-dimethylformamide (20 mL, 1.2 m) was added l-glutamine (3.47 g 23.7 mmol, 1 equiv) and the resulting solution was stirred at 90 °C for 16 hours. Complete consumption of starting materials was confirmed by TLC. The reaction mixture was allowed to reach room temperature and volatile components were removed *in vacuo*. The residue was re-dissolved in 4 m aqueous hydrochloric acid (20 mL, 1.2 m) and lithium chloride (1.0 g, 5% w/w) was added. After stirring of the reaction mixture at room temperature for a further 16 hours, the resulting precipitate was collected by filtration, washed with water and dried *in vacuo*, yielding phthalimide **14** (5.37 g, 18.3 mmol, 73%) as pale yellow amorphous solid.

Analytical data observed were as in accordance with literature values.^3^

^1^H NMR (400 MHz, methanol-*d_4_*) δ 7.87 (1H, ddd, *J* = 8.4, 7.4, 4.4 Hz, Ar-CH), 7.73 (1H, dd, *J* = 7.4, 0.7 Hz, Ar-CH), 7.55 (1H, ddd, *J* = 9.2, 8.4, 0.7 Hz, Ar-CH), 4.85 (1H, d, *J* = 4.8 Hz, -CH), 2.61 – 2.42 (2H, m, -CH_2_), 2.31 – 2.25 (2H, m, -CH_2_). ^13^C NMR (101 MHz, methanol-*d_4_*) δ 177.3 (-CONH_2_), 171.9 (-COOH), 168.0 (-CO), 165.8 (-CO), 159.0 (d, *^1^J_C-F_* = 263.8 Hz, Ar-C-F), 138.5 (d, *^3^J_C-F_* = 8.0 Hz, Ar-CH), 135.4 (Ar-C), 123.6 (d, *^2^J_C-F_* = 20.1 Hz, Ar-CH), 120.7 (d, *^4^J_C-F_* = 3.8 Hz, Ar-CH), 118.7 (d, *^2^J_C-F_* = 13.3 Hz, Ar-C), 52.9 (-CH), 32.9 (-CH_2_), 25.6 (-CH_2_). HRMS (ESI) exact mass calculated for C_13_H_11_FN_2_O_5_Na [M+Na]^+^ m/z 317.0544, found m/z 317.0551. LCMS (ESI) mass calculated for C_13_H_12_FN_2_O_5_ [M+H]^+^ m/z 295.07, found m/z 295.08 with *t*_R_ = 4.34 min.

**15**: To a stirred solution of phthalimide **14** (1.25 g, 4.25 mmol, 1 equiv) in acetonitrile (4 mL, 1 m) was added *N,N′-*carbonyldiimidazole (0.83 g, 5.1 mmol, 1.2 equiv) and 4-dimethylaminopyridine (20 mg, 0.18 mmol, 4 mol%), and the resulting solution was stirred under reflux whilst monitoring the reaction progress by TLC. After 6 hours the reaction mixture was allowed to cool to room temperature before removing volatile components *in vacuo*. The residue was re-dissolved in dichloromethane (25 mL) and water (25 mL), and the aqueous phase was extracted with dichloromethane (5 × 25 mL). The combined organic layers were dried over magnesium sulfate, filtered and concentrated *in vacuo*. Purification by column chromatography on silica gel with an eluent of 0% to 50% ethanol in ethyl acetate afforded **15** (647 mg, 2.35 mmol, 55%) as a yellow amorphous solid.

Analytical data observed were in accordance with literature values.^3^

^1^H NMR (400 MHz, chloroform-*d*) δ 8.02 (1H, s, -NH), 7.78 (1H, ddd, *J* = 8.2, 7.4, 4.3 Hz, Ar-CH), 7.72 (1H, dd, *J* = 7.3, 0.7 Hz, Ar-CH), 7.43 (1H, ddd, *J* = 9.2, 8.2, 0.7 Hz, Ar-CH), 5.02 – 4.95 (1H, m, -CH), 2.97 – 2.70 (3H, m, -C*H*H & -CH_2_), 2.20 – 2.12 (1H, m, -CH*H*). ^13^C NMR (101 MHz, chloroform-*d*) δ 170.8 (-CO), 167.8 (-CO), 166.3 (-CO), 164.1 (-CO), 158.1 (d, *^1^J_C-F_* = 267.2 Hz, Ar-C-F), 137.4 (d, *^3^J_C-F_* = 7.6 Hz, Ar-CH), 134.1 (Ar-C), 123.1 (d, *^2^J_C-F_* = 19.5 Hz, Ar-CH), 120.3 (d, *^4^J_C-F_* = 3.7 Hz, Ar-CH), 117.8 (Ar-C), 49.7 (-CH), 31.6 (-CH_2_), 22.8 (-CH_2_). HRMS (ESI) exact mass calculated for C_13_H_9_FN_2_O_4_Na [M+Na]^+^ m/z 299.0439, found m/z 299.0444. LCMS (ESI) mass calculated for C_14_H_14_FN_2_O_5_ [M+CH_3_OH+H]^+^ m/z 309.90, found m/z 309.92 with *t*_R_ = 5.05 min.

**16**: To a stirred solution of 2-[2-[2-(2-azidoethoxy)ethoxy]ethoxy]ethanol (1.0 g, 4.6 mmol, 1 equiv) in methanol (46 mL, 0.1 m) under an argon atmosphere was added 10% palladium on carbon (73 mg, 0.46 mmol, 10 mol%). The flask was evacuated and purged with argon three times, before sparging the suspension with hydrogen gas and stirring at room temperature under a hydrogen atmosphere for 16 hours. The hydrogen gas was removed by evacuating the flask and purging it with argon three times. After confirming complete consumption of starting material, the suspension was filtered through a pad of celite in dichloromethane and the celite washed with dichloromethane (250 mL) and methanol (250 mL). The solvents were removed *in vacuo* yielding aminoalcohol **16** (754 g, 3.90 mmol, 86%) as a pale yellow oil and the crude material was used in the next step without further purification.

**17**: To a stirred solution of aminoalcohol **16** (697 mg, 3.61 mmol, 1 equiv) in anhydrous dichloromethane (12 mL, 0.3 m) were added di-*tert*-butyl dicarbonate (945 mg, 4.33 mmol, 1.2 equiv) and triethylamine (0.75 mL, 5.4 mmol, 1.5 equiv) and the reaction mixture was stirred at room temperature for 16 hours. Volatile components were removed *in vacuo.* Purification by column chromatography on silica gel with ethyl acetate afforded protected aminoalcohol **17** (876 mg, 2.99 mmol, 83%) as a pale yellow oil.

Analytical data observed were in accordance with literature values.^4^

^1^H NMR (400 MHz, chloroform-*d*) δ 5.61 (1H, s, -NH), 3.75 – 3.49 (14H, m, 7 × -CH_2_), 3.30 (2H, t, *J* = 5.3 Hz, -CH_2_), 3.07 (1H, s, -OH), 1.42 (9H, s, 3 × -CH_3_). ^13^C NMR (101 MHz, chloroform-*d*) δ 156.3 (-NCOOR), 79.1 (-*C*(CH_3_)), 72.8 (-CH_2_), 70.8 (-CH_2_), 70.6 (2 × -CH_2_), 70.4 (-CH_2_), 70.2 (-CH_2_), 61.8 (-CH_2_), 40.5 (-CH_2_), 28.6 (3 × -CH_3_).

**18**: To a dry flask charged with argon was added anhydrous tetrahydrofuran (1.4 mL, 0.5 m), protected aminoalcohol **17** (200 mg, 0.68 mmol, 1 equiv) and sodium hydride (60% in mineral oil, 68 mg, 1.7 mmol, 2.5 equiv) at 0 °C. The resulting suspension was stirred for 30 minutes before 1-chloro-6-iodohexane (0.10 mL, 0.68 mmol, 1 equiv) was added slowly at 0 °C. The reaction mixture was allowed to reach room temperature and stirred for further 16 hours. The reaction was quenched by dropwise addition of water (10 mL). The aqueous phase was extracted with dichloromethane (3 × 10 mL) and the combined organic extracts were washed with brine (20 mL), dried over magnesium sulfate, filtered and concentrated *in vacuo*. Purification by column chromatography on silica gel with an eluent of 50 % ethyl acetate in petroleum ether afforded linker **18** (92 mg, 0.22 mmol, 33%) as a light yellow oil.

Analytical data observed were in accordance with literature values.^4^

^1^H NMR (400 MHz, chloroform-*d*) δ 5.04 (1H, s, -NH), 3.69 – 3.48 (16H, m, -CH_2_ & 7 × -CH_2_), 3.45 (2H, t, *J* = 6.6 Hz, -CH_2_), 3.35 – 3.25 (2H, m, -CH_2_), 1.80 – 1.72 (2H, m, -CH_2_), 1.63 – 1.55 (2H, m, -CH_2_), 1.43 (9H, s, 3 × -CH_3_), 1.40 – 1.32 (4H, m, 2 × -CH_2_). ^13^C NMR (101 MHz, chloroform-*d*) δ 156.1 (-NCOOR), 79.3 (-*C*(CH_3_)), 71.4 (-CH_2_), 70.8 (2 × -CH_2_), 70.8 (-CH_2_), 70.7 (-CH_2_), 70.4 (-CH_2_), 70.4 (-CH_2_), 70.3 (-CH_2_), 45.2 (-CH_2_), 40.5 (-CH_2_), 32.7 (-CH_2_), 29.6 (-CH_2_), 28.6 (3 × -CH_3_), 26.8 (-CH_2_), 25.6 (-CH_2_). IR (thin film) 3345, 1711, 864 cm^-1^. HRMS (ESI) exact mass calculated for C_19_H_38_ClNO_6_Na [M+Na]^+^ m/z 434.2280, found m/z 434.2265. LCMS (ESI) mass calculated for C_19_H_39_ClNO_6_ [M+H]^+^ m/z 412.25, found m/z 412.14 with *t*_R_ = 6.70 min.

**CRBN HaloPROTAC 2**: Protected linker **18** (62 mg, 0.15 mmol, 1 equiv) was dissolved in trifluoroacetic acid/dichloromethane (2.5 mL/2.5 mL, 0.03 m) and the resulting solution was stirred at room temperature for 3 hours. The reaction was monitored by TLC for complete consumption of the starting material before removal of volatile components *in vacuo*. The residue was redissolved in *N,N*-dimethylformamide (0.75 mL, 0.2 m), and CRBN ligand **15** (42 mg, 0.15 mmol, 1 equiv) and *N,N*-diisopropylethylamine (79 μL, 0.45 mmol, 3 equiv) were added. The resulting solution was heated to 90 °C and stirred for 16 hours. The reaction mixture was partitioned between dichloromethane (20 mL) and water (20 mL), and the aqueous phase was extracted with dichloromethane (3 × 20 mL). The combined organic phases were washed with 5% aqueous lithium chloride (3 × 20 mL), dried over magnesium sulfate, filtered and concentrated *in vacuo*. Purification by column chromatography on silica gel with an eluent of 25% to 100% ethyl acetate in dichloromethane, followed by reverse phase HPLC using a focused gradient across 50% and 90% acetonitrile in water over 30 minutes eluted the title compound at *t*_R_ = 27 minutes (81% acetonitrile). Lyophilisation of the product fraction generated **CRBN HaloPROTAC 2** (6 mg, 0.01 mmol, 7%) as a yellow fluffy powder.

Analytical data observed were in accordance with literature values.^4^

^1^H NMR (400 MHz, methanol-*d_4_*) δ 7.56 (1H, dd, *J* = 8.6, 7.1 Hz, Ar-CH), 7.13 – 7.04 (2H, m, 2 × Ar-CH), 5.09 – 5.02 (1H, m, -CH), 3.79 – 3.48 (18H, m, -CH_2_ & 8 × -CH_2_), 3.45 (2H, t, *J* = 6.5 Hz, -CH_2_), 2.91 – 2.66 (3H, m, -C*H*H & -CH_2_), 2.16 – 2.07 (1H, m, -CH*H*), 1.79 – 1.70 (2H, m, -CH_2_), 1.61 – 1.52 (2H, m, -CH_2_), 1.49 – 1.34 (4H, m, 2 × -CH_2_). ^13^C NMR (126 MHz, methanol-*d_4_*) δ 174.6 (-CO), 171.5 (-CO), 170.6 (-CO), 169.3 (-CO), 148.2 (Ar-C), 137.2 (Ar-C), 133.9 (Ar-CH), 118.3 (Ar-CH), 112.0 (Ar-CH), 111.3 (Ar-C), 72.1 (-CH_2_), 71.7 (-CH_2_), 71.7 (-CH_2_), 71.6 (-CH_2_), 71.6 (-CH_2_), 71.5 (-CH_2_), 71.2 (-CH_2_), 70.7 (-CH_2_), 50.2 (-CH), 45.7 (-CH_2_), 43.3 (-CH_2_), 33.8 (-CH_2_), 32.2 (-CH_2_), 30.5 (-CH_2_), 27.7 (-CH_2_), 26.5 (-CH_2_), 23.8 (-CH_2_). IR (thin film) 3387, 1697, 748 cm^-1^. HRMS (ESI) exact mass calculated for C_27_H_38_ClN_3_O_8_Na [M+Na]^+^ m/z 590.2240, found m/z 590.2220. LCMS (ESI) mass calculated for C_27_H_39_ClN_3_O_8_ [M+H]^+^ m/z 568.24, found m/z 568.67 with *t*_R_ = 6.42 min.

**References:**

(1) Buckley, D. L.; Raina, K.; Darricarrere, N.; Hines, J.; Gustafson, J. L.; Smith, I. E.; Miah, A. H.; Harling, J. D.; Crews, C. M. HaloPROTACS: Use of Small Molecule PROTACs to Induce Degradation of HaloTag Fusion Proteins. *ACS Chem Biol* **2015**, *10* (8), 1831-1837. DOI: 10.1021/acschembio.5b00442.

(2) Zoppi, V.; Hughes, S. J.; Maniaci, C.; Testa, A.; Gmaschitz, T.; Wieshofer, C.; Koegl, M.; Riching, K. M.; Daniels, D. L.; Spallarossa, A.; et al. Iterative Design and Optimization of Initially Inactive Proteolysis Targeting Chimeras (PROTACs) Identify VZ185 as a Potent, Fast, and Selective von Hippel–Lindau (VHL) Based Dual Degrader Probe of BRD9 and BRD7. *J Med Chem* **2019**, *62* (2), 699-726. DOI: 10.1021/acs.jmedchem.8b01413.

(3) Lu, J.; Qian, Y.; Altieri, M.; Dong, H.; Wang, J.; Raina, K.; Hines, J.; Winkler, J. D.; Crew, A. P.; Coleman, K.; et al. Hijacking the E3 Ubiquitin Ligase Cereblon to Efficiently Target BRD4. *Chem Biol* **2015**, *22* (6), 755-763. DOI: 10.1016/j.chembiol.2015.05.009 From NLM.

(4) Steinebach, C.; Sosič, I.; Lindner, S.; Bricelj, A.; Kohl, F.; Ng, Y. L. D.; Monschke, M.; Wagner, K. G.; Krönke, J.; Gütschow, M. A MedChem toolbox for cereblon-directed PROTACs. *Med Chem Comm* **2019**, *10* (6), 1037-1041, 10.1039/C9MD00185A. DOI: 10.1039/C9MD00185A.
