# Supplementary material for: Targeted degradation of zDHHC-PATs decreases substrate S-palmitoylation
Source: PLoS One. 2024 Mar 21;19(3):e0299665. doi: 10.1371/journal.pone.0299665 (PMC10956751; doi:10.1371/journal.pone.0299665)
Supplement: S2 File — (DOCX) [file pone.0299665.s003.docx]

VHL PROTAC 1 and epimer control 4:

**5**:

**6**:

**7a**:

**7b**:

**8a**:

**8b**:

**9a**:

**9b**:

**10**:

**11**:

**VHL HaloPROTAC 1:**

**Epimer control 4**:

VHL PROTAC 3:

**13**:

**VHL HaloPROTAC 3**:

CRBN PROTAC 2:

**14**:

**15**:

**17**:

**18**:

**CRBN HaloPROTAC 2**:
