# Supplementary material for: Targeted degradation of zDHHC-PATs decreases substrate S-palmitoylation
Source: PLoS One. 2024 Mar 21;19(3):e0299665. doi: 10.1371/journal.pone.0299665 (PMC10956751; doi:10.1371/journal.pone.0299665)
Supplement: Attachment — Submitted filename: Reviews & Responses.docx [file pone.0299665.s004.docx]

**Summary of Changes**

We are grateful to the reviewers and editor for their generally positive assessment of our manuscript. We particularly thank the reviewers for their suggestions to improve clarity and support our conclusions. We have undertaken new experiments and analysis to address most of the reviewers’ comments.

Figure 1:	Added new panel C demonstrating successful induction of HALO-tagged zDHHC-PATs using western blotting.

Figure 2:	Assessed off-target effects of compound 1 on un-tagged zDHHC-PATs by evaluating its impact on endogenous zDHHC5 (panel D).

Figure 5:	Added new data to panel B confirming successful induction of HALO-zDHHC-20 using western blotting.

**Editor Comments:**

When submitting your revision, we need you to address these additional requirements.

1. Please ensure that your manuscript meets PLOS ONE's style requirements, including those for file naming. The PLOS ONE style templates can be found at

https://journals.plos.org/plosone/s/file?id=wjVg/PLOSOne_formatting_sample_main_body.pdf and

https://journals.plos.org/plosone/s/file?id=ba62/PLOSOne_formatting_sample_title_authors_affiliations.pdf

Confirmed

2. Thank you for stating the following in the Acknowledgments Section of your manuscript:

"This work was supported by the British Heart Foundation, grant RG/17/15/33106 to MJS and WF and a Daphne Jackson Fellowship funded by Medical Research Scotland awarded to SS"

We note that you have provided funding information that is not currently declared in your Funding Statement. However, funding information should not appear in the Acknowledgments section or other areas of your manuscript. We will only publish funding information present in the Funding Statement section of the online submission form.

Please remove any funding-related text from the manuscript and let us know how you would like to update your Funding Statement. Currently, your Funding Statement reads as follows:

"This work was supported by the British Heart Foundation, grant RG/17/15/33106 "

Please include your amended statements within your cover letter; we will change the online submission form on your behalf.

Confirmed in the cover letter.

3. Thank you for stating the following financial disclosure:

"This work was supported by the British Heart Foundation, grant RG/17/15/33106 "

Please state what role the funders took in the study. If the funders had no role, please state: "The funders had no role in study design, data collection and analysis, decision to publish, or preparation of the manuscript."

If this statement is not correct you must amend it as needed.

Please include this amended Role of Funder statement in your cover letter; we will change the online submission form on your behalf.

Confirmed in the cover letter.

4. Please include captions for your Supporting Information files at the end of your manuscript, and update any in-text citations to match accordingly. Please see our Supporting Information guidelines for more information: http://journals.plos.org/plosone/s/supporting-information.

Confirmed.

5. PLOS ONE now requires that authors provide the original uncropped and unadjusted images underlying all blot or gel results reported in a submission’s figures or Supporting Information files. This policy and the journal’s other requirements for blot/gel reporting and figure preparation are described in detail at https://journals.plos.org/plosone/s/figures#loc-blot-and-gel-reporting-requirements and https://journals.plos.org/plosone/s/figures#loc-preparing-figures-from-image-files. When you submit your revised manuscript, please ensure that your figures adhere fully to these guidelines and provide the original underlying images for all blot or gel data reported in your submission. See the following link for instructions on providing the original image data: https://journals.plos.org/plosone/s/figures#loc-original-images-for-blots-and-gels.

In your cover letter, please note whether your blot/gel image data are in Supporting Information or posted at a public data repository, provide the repository URL if relevant, and provide specific details as to which raw blot/gel images, if any, are not available. Email us at plosone@plos.org if you have any questions.

Confirmed

6. Please review your reference list to ensure that it is complete and correct. If you have cited papers that have been retracted, please include the rationale for doing so in the manuscript text, or remove these references and replace them with relevant current references. Any changes to the reference list should be mentioned in the rebuttal letter that accompanies your revised manuscript. If you need to cite a retracted article, indicate the article’s retracted status in the References list and also include a citation and full reference for the retraction notice.

Confirmed

**Reviewer #1**: This manuscript presents data on a possible method to evaluate the integral membrane zDHHC-PAT enzymes to PROTAC mediated degradation. The authors report that zDHHC-PATs that are localized to both intracellular compartments and the cell surface membrane can be successfully degraded using PROTACs, thereby leading to decrease of substrate palmitoylation. Nonetheless there are a number of issues to be addressed:

1. P7, line 161~163, “by cereblon-directed Halo-PROTAC 2 (Figure 2b: Dmax 75±5% for compound 1, 61±13% for compound 3). In contrast all three Halo-PROTACs degraded Halo-zDHHC20 (Figure 2c: Dmax 78±6% for compound 1, 59±14% for compound 2, 60±10% for compound 3).” Please show the concentration of these Halo-PROTACs in the text.

We have added the information the reviewer requested, lines 175-177.

2. Figure 2C, this assay testified that degradation Halo-zDHHC20 in cells treated with compound 1 involves the polyubiquitination and subsequent proteasomal degradation of Halo-zDHHC20. One way to make the result more robust would be to set a control which add Tet and MG132 but without PROTAC compound 1; this might enable observation of a more convincing result.

We believe the reviewer is referring to Fig 3C. The reviewer is correct that this would tell us whether the proteasome is involved in the turnover of Halo-zDHHC20 in the absence of PROTAC. We highlight that the expression data provided in Figure 3B largely rules out a role for the proteasome in HALO-zDHHC turnover (identical expression of HALO-zDHHCs ± Mg-132). We have noted this information in the results, lines 197-198. Ultimately, even if HALO-zDHHC turnover is controlled by the proteasome, we don’t think this result would be particularly impactful (it would not be relevant for the endogenous zDHHCs – the system is too artificial to make this point worth chasing for now).

3. Figure 4. The reason for using Flot2 as an internal control should be mentioned and explained in the text.

We have added the information the reviewer requested to description of Figure 4, lies 228-230.

4. Vero E6 zDHHC20 knockout cell line should be verified by immunoblotting or sequencing.

This cell line was provided by a collaborator who published work confirming the absence of zDHHC20. We have added this information to the results section, line 274.

5. P10, line 254~255, “but we were unable to degrade Halo-zDHHC20 with any Halo-PROTACs (Figure 5b).” However, in figure 5B, it seems that compound 1 significantly decrease the Halo-zDHHC20 level at the concentration of 1µM in WB result.

We apologise. On average 1µM did not have any meaningful impact on expression, but we picked a bad representative blot and have replaced it with an alternative replicate.

6. In Figure 5C, the results shown in the chart are not very consistent with those in WB. Obviously, in WB result, the verapamil enhanced compound 1 efficacy on Halo-zDHHC20 degradation more potent at 24 hours than that at 48 hours.

We apologise – we picked a bad representative blot and have replaced it with an alternative replicate.

**Reviewer #2**: This is a review of the paper “Targeted degradation of zDHHC-PATs decreases substrate S-palmitoylation” for PLOS ONE.

This paper points out that the inhibition of the activity of individual zDHHC-PATs is reasonable, but the specificity of existing tools is poor. Thus, the authors developed 3 molecules based on PROTACs to target this class of enzymes. They evaluated the impact of Halo-PROTACs on zDHHC-PAT expression and substrate palmitoylation. While, they concluded that although PROTAC-mediated targeting of zDHHC-PATs to decrease substrate palmitoylation is feasible, given the well-established degeneracy in the zDHHC-PAT family, in some settings the activity of non-targeted zDHHC-PATs may substitute and preserve substrate palmitoylation.

Some changes that are recommended.

1. Figure 1, the author only detected tetracycline induced protein expression by Confocal microscopy. However, before this assay, I advised they could detect the total protein and mRNA expression levels by Western blot and qPCR.

Thank you for the suggestion. We have added Western blots to Figure 1 and Figure 5 to demonstrate that the cell lines used express a Halo-tagged protein of the predicted molecular weight in a tetracycline-inducible manner.

2. It is not necessary to show the protein marker in the WB pictures, the author should clip the original image and make them showed in general types, such as cropped gel band, band molecular weight, cell lines name, band’s name labeled in the left of the panel.

We have cropped off all the ladders as suggested, except for a couple of examples where the ladder helps the reader understand the experiment better (ubiquitination experiment, spike protein).

3. Line 250-252, the authors should provide data that proved the endogenous zDHHC20 was knockout.

This cell line was provided by a collaborator who published work confirming the absence of zDHHC20. We have added this information to the results section, line 274.

4. It is recommended that introduce the Halo-PROTACs technology and its application in zDHHC-PATs degradation in the introduction chapter.

Thank you for the recommendation. We have added the information the reviewer suggests. See lines 117-121.

5. The author should detect the cell toxicity of these compound.

We did not formally test toxicity of the Halo-PROTACs but we observed no impact on cell growth or morphology using them up to 10µM (10x higher than the highest concentration reported in our investigation). These compounds have been widely used in the literature and others have already reported no toxicity when used below 10µM (ACS Chem Biol 10, 1831-1837 (2015)). We have added this information to the introduction, lines 117-121.

6. The writing in the Methods chapter is overly simplistic and many important details are not clearly written.

We have rewritten several sections of the methods to improve clarity.

7. The author should perform additional assay to prove the specificity of compound 1.

Thank you for the suggestion. We evaluated whether compound 1 impacts expression of endogenous zDHHC-PATs by probing for zDHHC5 in cells treated with compound 1. There is no impact – the data is presented in Figure 2d and lines 182-183. We cannot do the same for zDHHC20 because the antibodies for the endogenous protein are unreliable in our hands. The striking specificity of Halo-PROTACs at a whole proteome level has been described by others, which we now mention in the introduction (lines 117-121).

Overall, after making corrections and additions, I think this is a useful paper to publish.
